# Generation and characterization of iPSC-derived renal proximal tubule-like cells with extended stability

**DOI:** 10.1038/s41598-021-89550-4

**Published:** 2021-06-02

**Authors:** Vidya Chandrasekaran, Giada Carta, Daniel da Costa Pereira, Rajinder Gupta, Cormac Murphy, Elisabeth Feifel, Georg Kern, Judith Lechner, Anna Lina Cavallo, Shailesh Gupta, Florian Caiment, Jos C. S. Kleinjans, Gerhard Gstraunthaler, Paul Jennings, Anja Wilmes

**Affiliations:** 1 Division of Molecular and Computational Toxicology, Department of Chemistry and Pharmaceutical Sciences, Vrije Universiteit Amsterdam, De Boelelaan 1108, 1081 HZ Amsterdam, The Netherlands; 2 Department of Toxicogenomics, Maastricht University, School of Oncology and Developmental Biology (GROW), Maastricht, The Netherlands; 3 Institute of Physiology and Medical Physics, Medical University of Innsbruck, Innsbruck, Austria; 4 Discovery Sciences R&D, AstraZeneca, Gothenburg, Sweden

**Keywords:** Cell biology, Stem cells

## Abstract

The renal proximal tubule is responsible for re-absorption of the majority of the glomerular filtrate and its proper function is necessary for whole-body homeostasis. Aging, certain diseases and chemical-induced toxicity are factors that contribute to proximal tubule injury and chronic kidney disease progression. To better understand these processes, it would be advantageous to generate renal tissues from human induced pluripotent stem cells (iPSC). Here, we report the differentiation and characterization of iPSC lines into proximal tubular-like cells (PTL). The protocol is a step wise exposure of small molecules and growth factors, including the GSK3 inhibitor (CHIR99021), the retinoic acid receptor activator (TTNPB), FGF9 and EGF, to drive iPSC to PTL via cell stages representing characteristics of early stages of renal development. Genome-wide RNA sequencing showed that PTL clustered within a kidney phenotype. PTL expressed proximal tubular-specific markers, including megalin (LRP2), showed a polarized phenotype, and were responsive to parathyroid hormone. PTL could take up albumin and exhibited ABCB1 transport activity. The phenotype was stable for up to 7 days and was maintained after passaging. This protocol will form the basis of an optimized strategy for molecular investigations using iPSC derived PTL.

## Introduction

Chronic kidney disease (CKD) has a prevalence in Europe ranging from 3 to 17%^1^, which is associated with increasing incidence of diabetes and cardiovascular disease as well as increasing lifespans^2,3^. Furthermore, exposure to nephrotoxic compounds is likely to accelerate CKD progression. The kidney receives approximately 20% of the cardiac output and is vital in the elimination of xenobiotics. The cells of the nephron exhibit a high degree of physiological, morphological, and biochemical heterogeneity. Due to the discrete properties of the individual cells and their immediate environment, individual nephron segments exhibit site-specific sensitivities to xenobiotics, primarily due to region-specific external and/or internal xenobiotic concentrations. Of these, the glomerulus and the proximal tubule are the most frequent nephron regions adversely affected by xenobiotics and thus are the most studied in the context of drug safety assessment.

Over recent years a number of human in vitro model systems have been shown to be useful for their applicability to mechanism driven toxicity assessment by employing integrated systems biology methods and biokinetics approaches^4–6^. For example, the hTERT-immortalized human proximal tubular cell line RPTEC/TERT1^7,8^ has been employed to identify mechanistic and functional aspects of xenobiotic exposure^9–13^. However, a major limitation of using cell lines is the fact that they are derived from a single donor and thus represent only a single genetic background. The discovery of a method to induce a pluripotent stem cell status in any somatic cell^14^ provides the possibility of expanding in vitro toxicological investigations to entire populations^15^. Protocols to differentiate induced Pluripotent Stem Cells (iPSCs) into various cell lineages for toxicological applications including neurons, cardiomyocytes, hepatocytes and renal cell types have been established^15–18^.

Several groups have reported the generation of three-dimensional self-organizing kidney organoids structures from iPSC^19–23^. iPSC-derived renal organoids contain nephron-like structures, including glomerular structures, proximal tubular cells, loop of Henle cells, distal tubular cells and in some protocols collecting duct structures, in a highly structured and organized way. Renal organoids offer exciting opportunities in the fields of regenerative medicine and disease modelling and to some degree in toxicity assessment. However, there are also several limitations associated with renal organoids, including the lack of a vascular system^24^, contamination with non-renal cells (including up to 20% neurons and muscle cells)^25^ and lack of maturity of cell types present. In addition, toxicity testing in renal organoids is challenging for several reasons, e.g. it is difficult to expose the cells exclusively from either apical or basolateral side and to evaluate how much compound each cell type of the organoid is exposed to. It is also more challenging to analyze specific effects on individual cell types present in the organoid. Furthermore, the time to generate and complexity of the renal organoids make them currently less amenable for high through-put applications. However, there has been some progress to increase through-put by generating smaller so-called micro-organoids^26^. Monocultures of a target cell type could overcome some of these shortcomings. Recently, several protocols to drive iPSC directly into renal podocytes have been developed^27–31^. However, few protocols have been described to drive iPSC directly into renal proximal tubular cells^32^, and the expression of the proximal specific protein megalin and long-term stability have not been reported in these cells. Megalin is a large endocytic receptor that is highly expressed in the renal proximal tubule and together with cubilin it is responsible for the reabsorption of filtered proteins and several other ligands from the glomerular filtrate^33^.

Here, we developed a rapid and simple protocol to drive iPSC into proximal tubular like cells (PTL) within 14 days using temporal addition of a combination of small molecules and growth factors. The progression of the iPS cells to PTL was analyzed using RNA sequencing (RNASeq) and protein expression. Some functional assays were also used for characterization including albumin uptake, ABCB1 mediated extrusion and hormonal sensitivity.

## Results

### Differentiation of PTL

**Figure 1:**
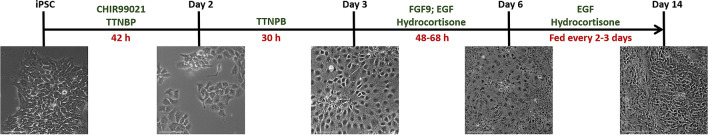
Schematic representation of the protocol to differentiate induced human Pluripotent Stem Cells (iPSC) into renal proximal tubule like (PTL) cells. Human iPSC were seeded on Geltrex coated cell culture wells and incubated with the indicated growth factors and chemicals in sequential steps for 14 days. Images were captured with a phase contrast microscope. The white scale bar represents 100 µm.

A scheme of the protocol for differentiation is given in Fig. 1. iPSC colonies (day 0) were detached and seeded into single cells. By day 3, cells appeared cobblestone-like and formed a confluent monolayer (Fig. 1). After treatment with FGF9, some cell death was observed at day 6. The monolayer recovered in the next step without FGF9. Differentiation of PTL could be performed on both, Geltrex-coated plates and RPTEC/TERT1 ECM-coated plates with equal efficiency. No major differences between the two coatings were observed, although an extensive comparison between both coatings was not conducted in the current study.

### Characterization of differentiation markers of PTL using a genome wide RNASeq

**Figure 2:**
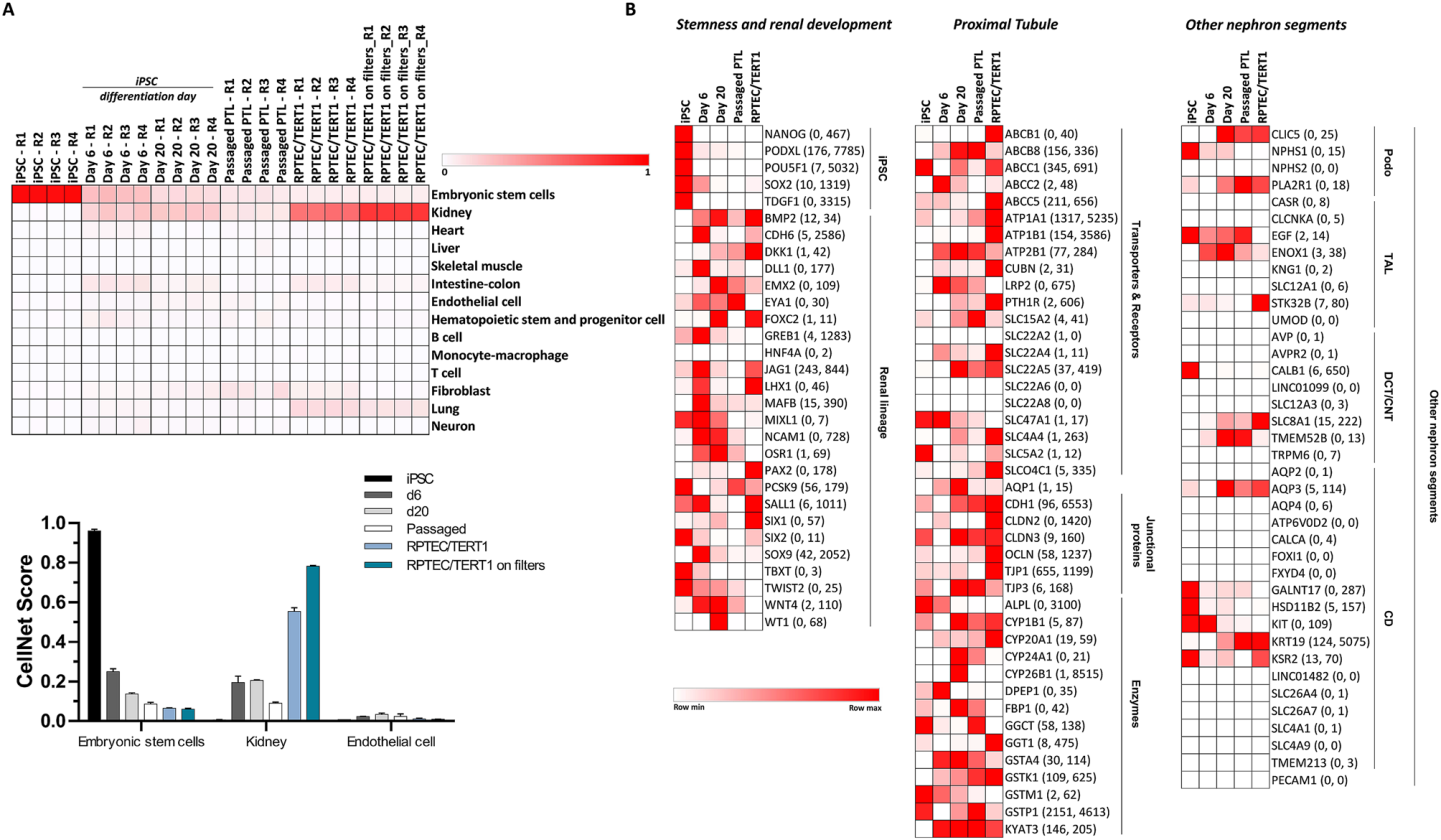
Heat map representation of mRNA expression quantified by RNASeq from selected genes in iPSC, PTL differentiations and RPTEC/TERT1 cells. iPSCs (SBAD3) were differentiated into PTL on ECM for 20 days. RNASeq analysis was performed on samples collected from undifferentiated iPSC, day 6 and 20 after differentiation and PTL passaged at day 16 of differentiation and cultured for 4 additional days after passaging. RPTEC/TERT1 cells were used for comparison. Results are presented for 4 independent replicates (R1 to R4). Heatmaps were generated by Microsoft Excel 365 conditional formatting with a 2-scale colour system (white (low) to red (high)). (**A**) CellNet (Cahan *et al*. 2014) was used to compare sample transcripts with existing tissue data bases. The higher the similarity, the higher the score. Scaling is conducted across all samples and tissues. The mean CellNet score ± standard deviation is plotted in the graph. (**B**) Heat maps represent read count expression. The abbreviation represents the official gene symbol. The different gene subsets are sorted in alphabetical order. Minimum and maximum normalised read counts are given in parenthesis. Scaling is conducted per gene.

RNASeq was performed on the iPSC line (SBAD3) at four different conditions: (1) prior to differentiation (undifferentiated iPSC), (2) at day 6, (3) at day 20 and (4) after one passage (passaged PTL). The commonly used differentiated human proximal tubule cell line RPTEC/TERT1 cells were used as a comparison. RNASeq data was analyzed using CellNet^34^, which is a biology-based computational platform to determine cell type–specific gene regulatory networks (GRN) from gene expression profiles, as well as literature-based analysis comparing known markers of undifferentiated iPSC, renal developmental and renal proximal tubular markers. As a qualification, CellNet correctly mapped undifferentiated iPSC cells with embryonic stem cells (ESC, 0.96 ± 0.009) and RPTEC/TERT1 cells with the kidney (0.55 ± 0.019 and 0.78 ± 0.005), for plastic and filter cultured cells, respectively, Fig. 2A). iPSC differentiation at day 6 showed clustering with both, the kidney (0.19 ± 0.033) and ESC (0.25 ± 0.015). At day 20 there was less ESC clustering (0.14 ± 0.006) and a slightly stronger similarity with the kidney dataset (0.21 ± 0.003). Passaged PTL also clustered with the kidney dataset, but to a lesser degree (0.09 ± 0.005). However, for this particular experiment cells were sub-cultured for only 4 days, the similarity might have been improved if we had left them longer.

RNASeq data was also analyzed on an individual gene level using selected markers from literature, from single cell RNASeq data from human kidney tissue^35^ and from the LifeMap^36^ (discovery.lifemapsc.com) and Protein Atlas^37^ (proteinatlas.org) data bases. Undifferentiated iPSC expressed high levels of pluripotent (stemness) markers, including NANOG, PODXL (aka Tra-1-60), POU5F1 (aka Oct3/4) and SOX2 (Fig. 2B, iPSC). For stemness markers, by day 6, expression of NANOG and POU5F1 were undetectable. Levels of PODXL and SOX2 decreased at day 6 and were further reduced by day 20. For renal development markers, at day 6, several markers were highly expressed, including, SALL1, CDH6, DLL1, GREB1, JAG1 and SOX9. Other renal developmental markers were present at lower levels, including, OSR1, PAX2, EYA1, BMP2, LHX1 and DKK1 (Fig. 2B, Renal lineage). By day 20, most of these markers, including CDH6, DLL1, GREB1 were reduced, whereas some markers sustained a high expression level on day 20, including EMX2, NCAM and DKK1.

With respect to genes expected to be enriched in the proximal tubule nephron, the LRP2 gene which encodes megalin was highly expressed in PTL at days 6 and 20. RPTEC/TERT1 cells showed no LRP2 expression (Fig. 2B). Markers that are highly expressed in the proximal tubule part of the nephron in vivo, but also in a broader variety of other tissues, include PTH1R (parathyroid hormone receptor 1), AQP1 (aquaporin 1) and the tight junction proteins CLDN2 (claudin 2) and tight junction protein 3 (TJP3 aka ZO3). PTH1R was expressed in PTL at day 20 and passaged PTL, AQP1 was highest at day 20 PTL and CLDN2 was only expressed at day 6 (Fig. 2B). TJP3 was expressed in iPSC, decreased at day 6 and increased at day 20 and passaged cells. RPTEC/TERT1 were also positive for TJP3. Transporters and receptors typically associated to the proximal tubule which were enriched in differentiated cells included ABCC1 (MRP1) and ABCC2 (MRP2), SLC22A4 (OCTN1), SLC22A5 (OCTN2), the sodium bicarbonate cotransporter (SLC4A4) and SLC15A2 (PEPT2). Transcripts of organic anion transporters (OAT1, OAT3) and organic cation transporters (OCT2) could not be detected in PTL or RPTEC/TERT1 cells with RNASeq in this study. Nevertheless, the protein expression of OCT2, OAT1 and OAT3 was previously described in RPTEC/TERT1 cells^9^. Transcripts of enzymes that were expressed in PTL include fructose 1,6-bisphospahase (FBP1), KYAT3 (Kynurenine—oxoglutarate transaminases / Cysteine-S-conjugate beta-lyase 2), glutathione S-transferase isoforms (GSTs) as well as the cytochrome P450 enzymes CYP1B1, CYP24A1 and CYP26B1. CYP24A1 is part of the 25-hydroxyvitamin D3-24-hydroxylase enzyme that is involved in hydroxylation of 25-hydroxyvitamin D3 and 1,25-dihydroxyvitamin D3, and is the key enzyme in Vitamin D catabolism^38^. It is expressed in the kidney as well as in other tissues expressing the Vitamin D receptor^38^. CYP26B1 has a wide tissue distribution, including the liver, lung and kidney where it is involved in retinoic acid metabolism^39^. CYP1B1 is constitutively expressed in several extrahepatic tissues, including the kidney^40^ and expression of CYP1B1 has previously been reported in freshly isolated human primary proximal tubular cells and in RPTEC/TERT1 cells^41,42^. RPTEC/TERT1 cells in this study also showed expression of CYP1B1 (Fig. 2B). Finally, genes expressed preferentially in other nephron segments than the proximal tubular are shown in Fig. 2B (other nephron segments). These were identified from a single cell RNASeq study of human kidney tissue^35^. mRNA of most of these markers was either absent or expressed at low levels in the PTL (Fig. 2B), including the podocyte marker NPHS2, the TAL markers UMOD and SLC12A1, the distal convoluted tubule marker and connecting tubule markers SLC12A3 and CALB1 and the collecting duct markers AQP2, ATP6V0D2, SLC26A7 and SLC26A4.

In summary, the RNASeq study showed that PTL resembled more a kidney tissue than other tissues and expressed certain, but not all, expected markers for renal proximal tubular cells. RPTEC/TERT1 cells also showed a similarity to kidney tissue, which was better on filter cultured cells. However, several proximal tubular genes, including LRP2, FBP1 and CYP24A1 were only expressed in PTL and not in RPTEC/TERT1 cells.

### Expression of differentiation markers via immunofluorescence

**Figure 3:**
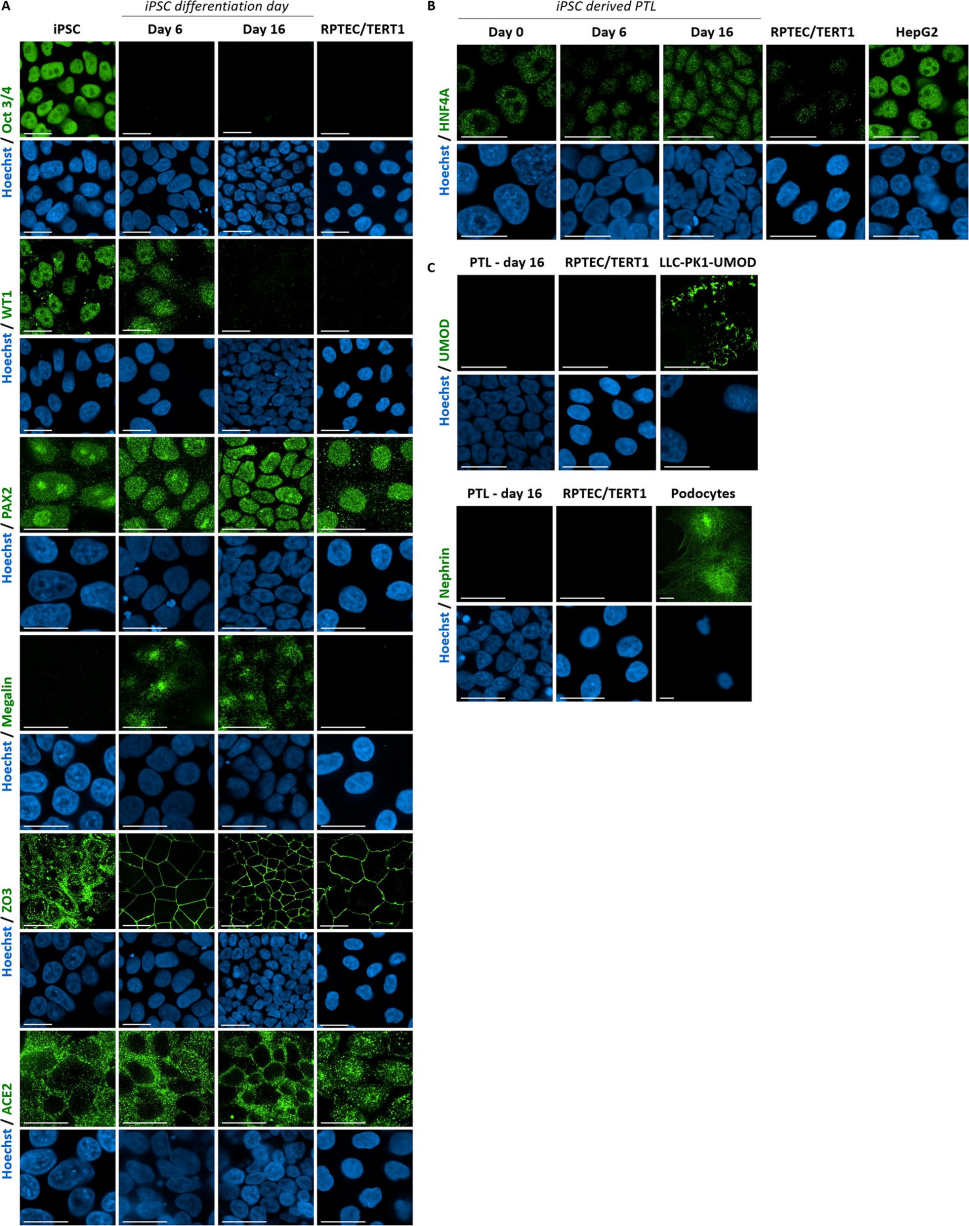
Immunofluorescence imaging of PTL and control cell lines. iPSC (SBAD2) were differentiated on Geltrex in 96 well pates as indicated and fixed at day 0, day 6 and day 16. Indicated immune targets are given in green and Hoechst staining is shown in blue (**A**). Images were captured using confocal microscopy with 40 × or 63 × water objectives. All exposure settings were the same. The scale bar represents 20 µm. HepG2, human uromodulin expressing (transgenic) LLC-PK1 cells and iPSC derived podocytes were used as positive controls for HNF4A (**B**), uromodulin and nephrin (**C**) respectively. The images are scaled post acquisition to the positive control. (The podocyte image is non-confocal).

Differentiation markers of different stages during renal development were characterized in three different iPSC lines. Oct3/4 (aka POU5F1) staining was visualized in the nuclei in iPSC (day 0) and was absent after differentiation (Fig. 3A, Supplementary Fig. S1). The renal developmental marker Wilm’s tumor 1 (WT1) showed nuclear expression in iPSC and on day 6 after differentiation but was absent on day 20. PAX2 demonstrated nuclear expression in all stages and in RPTEC/TERT1 cells. Megalin (LRP2) was not expressed in iPSC or RPTEC/TERT1 cells but was observed at day 6 of and on day 16 (Fig. 3A, Supplementary Fig. S1). Tight junction protein 3 (ZO3, TJP3) showed a nuclear staining in iPSC and a membrane staining on days 6 and 16 after differentiation into PTL (Fig. 3A, Supplementary Fig. S1). Confluent iPSC also showed membrane localization of ZO3 (not shown). Angiotensin-converting enzyme (ACE2) staining could be detected in iPSC, PTL and in RPTEC/TERT1 cells (Fig. 3A, Supplementary Fig. 1). Nuclear staining of hepatocyte nuclear factor 4 alpha (HNF4A) could be detected at low levels in iPSC and in RPTEC/TERT1 cells and at higher levels in PTL at day 16 (Fig. 3A,B, Supplementary Fig. S1). HepG2 were used as a positive control and showed the highest levels of HNF4A from across all tested cell types (Fig. 3B). Selected non-proximal tubular markers, uromodulin (UMOD, thick ascending limb) and nephrin (glomerular podocytes), showed no staining in PTL or RPTEC/TERT1 (Fig. 3C). Positive control cells showed the expected reactivity.

### Expression of differentiation markers via western blot analysis

**Figure 4:**
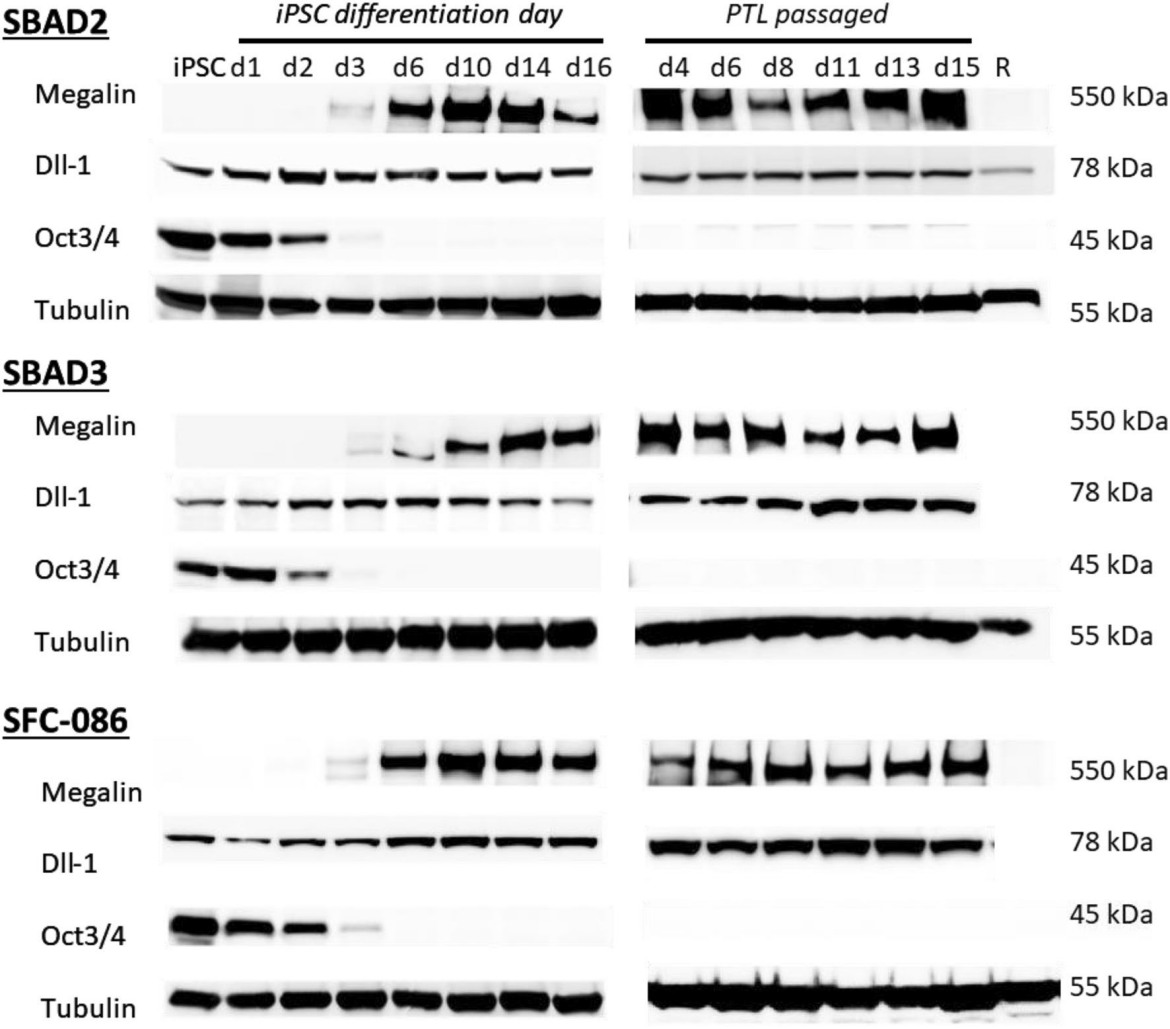
Western blot of time course of protein expression of stemness and differentiation markers. The iPSC lines SBAD2, SBAD3 and SFC-086 were differentiated on ECM as indicated. Cells were lysed at various time points during differentiation course. PTL were passaged on day 16 and grown for 15 further days. For each cell line at least 3 independent differentiations were performed. RPTEC/TERT1 cell lysates (R) were ran as a comparison. Samples were run on gradient gels and transferred to PVDF membranes using a semi-dry transfer.

Megalin was absent in iPSC and on the first two days of differentiation. Megalin expression was shown to increase from day 3 of differentiation and maintained at least until day 16 with maximum level shown around day 10 to day 14. (Fig. 4). DLL1, was expressed at all investigated time points and was also expressed in RPTEC/TERT1 cells. Oct3/4 (aka POU5F1) was highly expressed in iPSC prior to differentiation, decreased on days 1 and was absent by day 6 (Fig. 4). In passaged conditions megalin expression was confirmed up to 15 days in all 3 donors (Fig. 4).

### Characterization of passaged PTL

**Figure 5:**
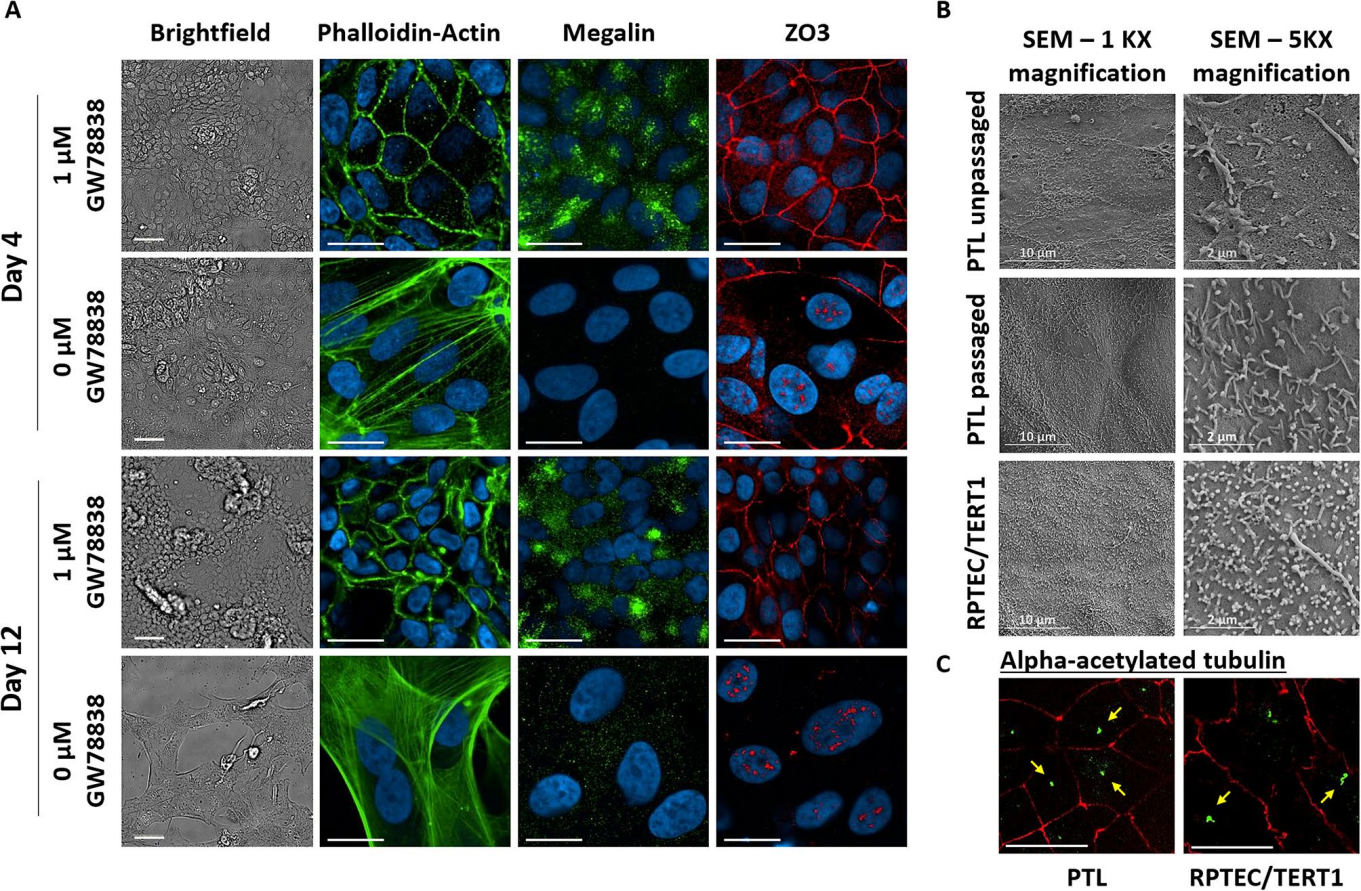
Light, electron and immunofluorescent microscopy images showing morphology of passaged and unpassed PTLs. (**A**) iPSC (SBAD2) were differentiated on Geltrex as indicated. On day 16 cells were passaged and plated on Geltrex in the presence or absence of 1 µM GW788388 hydrate. On day 4 and 12 cells were fixed in 4% PFA and stained with phalloidin, Hoechst and antibodies against megalin and ZO3. (**B**) For unpassaged cells, iPSC (SBAD3) were differentiated on 24 well transwell filters for 16 days. Passaged cells were differentiated on 6 well plastic dishes for 16 days and passaged onto transwell filters and cultured for 10 days. RPTEC/TERT1 were cultured on transwell filters for comparison. Samples were fixed with glutaraldehyde/PFA solution and processed for SEM images. (**C**) PTL and RPTEC/TERT1 cultured on 96 well plates were fixed and stained with alpha-acetylated tubulin (green: pointed out by yellow arrow) along with occludin (red). Images from different z-planes were combined. Scale bars are 20 µm unless stated otherwise.

Here, we developed a protocol that allowed passaging and freeze-thawing of PTL at least once. One essential requirement for maintaining the cells in a differentiated and polarized phenotype after passaging was the addition of GW788388 hydrate, a selective TGF-beta receptor inhibitor^43^ (Fig. 5A). In the absence of GW788388 hydrate cells quickly changed their morphology from cobblestone shaped cells to a fibroblast-like morphology within 4 days in culture, actin staining showed a non-epithelial like distribution and ZO3 (TJP3) was expressed in the nuclei (Fig. 5A). Within 7 days of culture, most of the cells died and detached from the dish when passaged PTL were cultured without addition of GW788388 hydrate (data not shown) and remaining attached cells lost their polarized phenotype (Fig. 5A). In contrast, in the presence of 1 µM GW788388 hydrate, cells remained a polarized phenotype for at least up to 12 days after passage (Fig. 5A). In addition, different coating methods for seeding the passaged PTL were tested, including collagen IV coating, ECM-coating, Geltrex coating or no coating at all. We could show that all four coatings maintained polarized PTL, including uncoated plates (data not shown), suggesting that matured PTL produce and release their own ECM, as RPTEC/TERT1 cells (unpublished observation). It was important not to dilute the cells too much during seeding, as when they were seeded too low, they stopped proliferating before reaching confluence and thus did not form an intact epithelial monolayer. A seeding density of 2.5 to 5 × 10^5^ cell/ml was deemed acceptable. Scanning electron microscopy (SEM) analysis of differentiated PTL confirmed a monolayer formation and showed brush border structures, including microvilli at the apical surface that were similar to brush border features observed in RPTEC/TERT1 (Fig. 5B, Supplementary Figure S2). Furthermore, cilia could be detected in some SEM images and was confirmed in both cell types by staining with acetylated tubulin (Fig. 5C).

### Assessment of functional parameters of PTL

**Figure 6:**
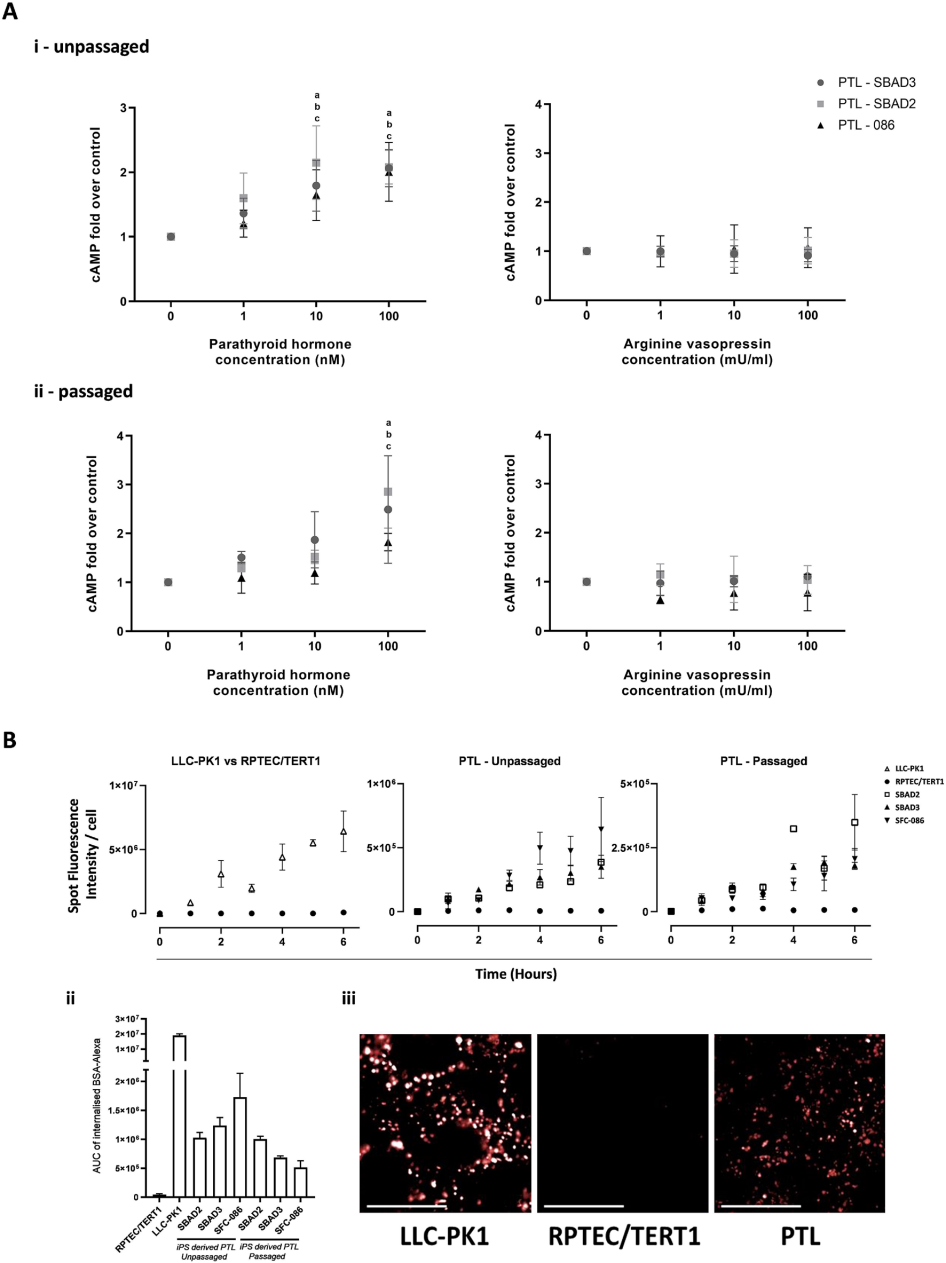
Functional characterisation of PTL. (**A**) Cellular cAMP response to parathyroid hormone (PTH) and arginine vasopressin (AVP). Three different iPSC lines (SBAD2, SBAD3 and 086) were differentiated on ECM until day 16 or passaged on day 16 and cultured further for 11 days. Cells were treated with different concentrations of AVP and PTH for 20 min. cAMP levels were quantified with a competitive EIA and values were normalised to IBMX controls. Data points represent the mean of at least 3 independent experiments plus and minus the standard deviation. Statistical analysis was conducted via a one-way ANOVA. a, b and c represent statistical relevance compared to control (P < 0.05), for SBAD2, SBAD3 and SFC-086 PTLs respectively. (**B**) Albumin uptake assay. iPSC derived PTLs cultured on Geltrex, RPTEC/TERT1 and LLC-PK1 were treated with 10 µg/ml BSA-Alexa-647. Images were captured using Operetta high content imager (63X water objective). The endocytosis of labelled BSA was observed as district fluorescent spots which was quantified over time (Bi) and represented as Area Under the Curve (AUC) in B-ii. Representative images are given in B-iii. Scale bar represents 20 µm.

In the proximal tubule, exposure of parathyroid hormone (PTH) leads to activation of adenylate cyclase and a subsequent increase in cyclic AMP (cAMP)^44^. This is mediated by the receptor PTH1R. In the collecting duct, activation of adenylate cyclase and subsequent increases in cAMP levels is observed in response to arginine vasopressin (AVP) exposure and is mediated by the receptor AVPR2. In this study, expression of PTH1R but not AVPR2 could be detected in PTL (Fig. 2B). PTH and AVP response was examined in the three different iPSC lines (SBAD2, SBAD3 and SFC086) on day 16 after differentiation into PTL and on day 10 in passaged PTL cells. PTL were incubated with different concentrations of PTH and AVP in the presence of 100 µM IBMX. PTH, but not AVP induced cAMP levels in a dose dependent manner in matured PTL and in matured and passaged PTL (Fig. 6A). This was similar to the results previously reported in the RPTEC/TERT1 cells^7^, suggesting that both cell types, show a proximal tubule and not distal / collecting duct hormonal response.

Megalin is responsible for the reabsorption of albumin and other proteins from the glomerular filtrate via receptor-mediated endocytosis. Megalin is expressed on the apical side of proximal tubule epithelial cells. Here, we tested whether megalin-expressing PTL facilitate the uptake of fluorescent-labelled albumin. The PTL were compared to the megalin-expressing LLC-PK1 cells (porcine proximal tubular cell line, positive control)^45^ and to the RPTEC/TERT1 cell line (lacking megalin expression, negative control). LLC-PK1 cells showed the highest amount of albumin uptake, whereas RPTEC/TERT1 cells did not show any significant uptake (Fig. 6B). Both, unpassaged and passaged PTL, showed a clear uptake of labelled albumin (Fig. 6B).

**Figure 7:**
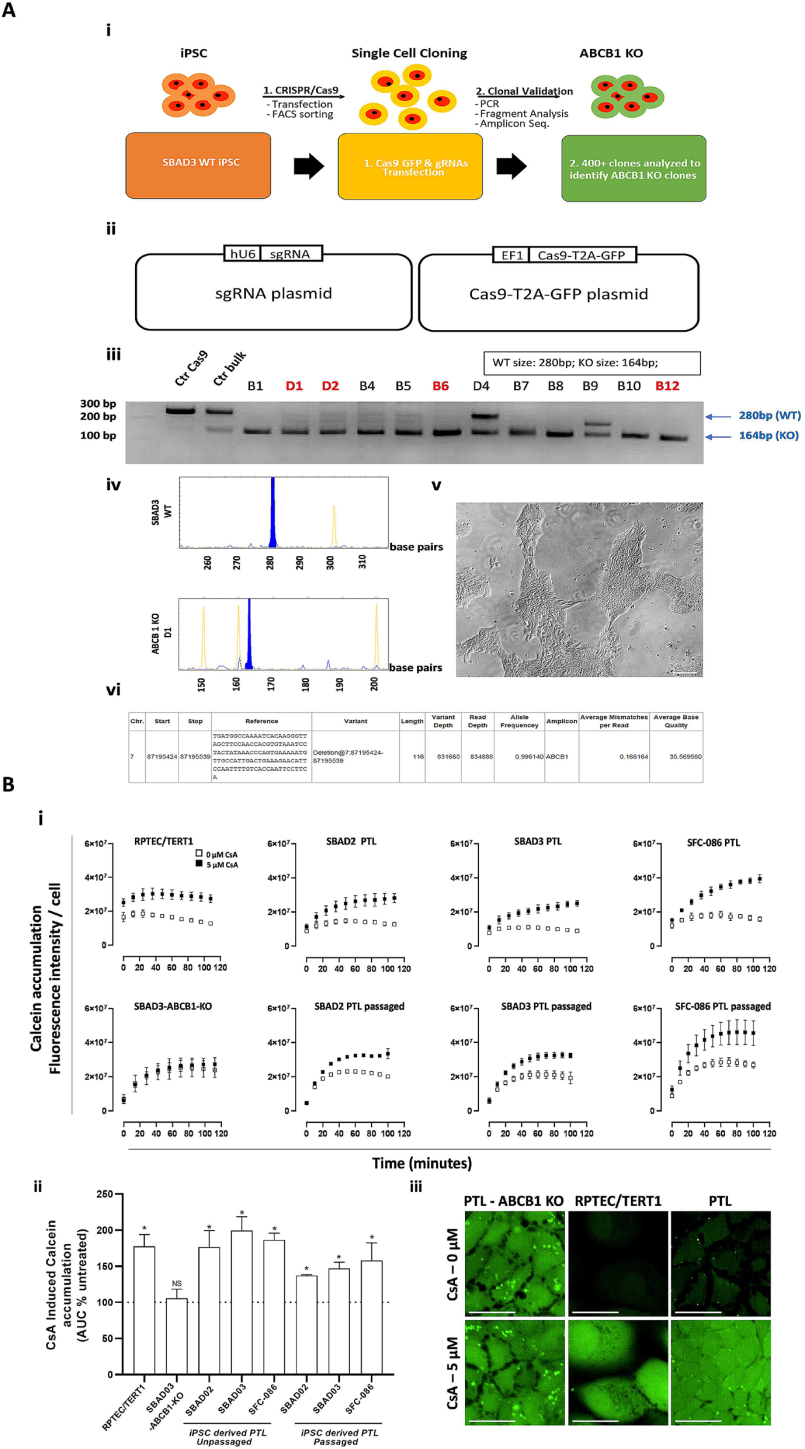
Characterisation of calcein-AM efflux in PTL. (**A**) Generation of ABCB1 KO iPSC. (A-i) Schematic of strategy & work flow for generation of ABCB1 KO iPS cell line. ABCB1 KO iPS cell line was generated using StemBANCC hiPSC line SBAD3 clone 01. (A-ii) Schematics of sgRNA and Cas9-T2A-GFP CRISPR plasmids. (A-iii) PCR analysis identified clones with ABCB1 (116 bp deletion) mutation. Clone D1 was selected for further analysis. (A-iv) Fragment analysis of PCR gel from SBAD3 Wild Type (WT) cells with a peak at 280 bp and ABCB1 KO-D1 clone with a peak at 164 bp (Y axis represents DNA base pairs). (A-v) Representative bright field image of live culture of clone D1 (scale bar = 200 µM). (A-vi) Next generation sequencing analysis through amplicon sequencing of D1 clone shows 116 bp deletion in ABCB1 gene. (**B**) Characterization of calcein-AM efflux in the D1 clone. (B-i) Quantification of calcein accumulation in cells with (closed squares) and without 5 µM CsA (open squares). RPTEC/TERT1 cells (top panel, left) served as a positive control and PTLs derived from SBAD3-ABCB1-KO clone D1 (bottom panel, left) served as a specificity control. PTL from 3 different donors were tested at either unpassaged state (top panel, 2–4) or passaged state (bottom panel, 2–4) (B-ii) Area under the curve of kinetic curves is expressed as percentage of corresponding untreated samples. A two tailed unpaired Student’s t-test was performed between AUCs of untreated and CsA treated populations, *P* value cut off is *P* < 0.05. (B-iii) Representative images of calcein accumulation of ABCB1 KO PTL cells (negative control), RPTEC/TERT1 cells (positive control) and PTL are shown in the presence and absence of the inhibitor CsA. Scale bar represents 20 µm.

P-glycoprotein (Pgp, ABCB1), is an important xenobiotic efflux transporter. Pgp is present in numerous tissues with high expression levels in the liver, kidney and blood brain barrier. In the nephron, Pgp expression is highest in the proximal tubule, where it is localized on the apical side and is involved in the clearance of substrates from the epithelial cells. Here, we investigated whether PTL showed active transport of the Pgp substrate calcein acetoxymethyl ester (calcein-AM). Calcein-AM is rapidly hydrolysed in the cells to form the fluorescent product calcein, which itself is not a Pgp substrate. Cyclosporine A (CsA), a potent Pgp substrate was used as a completive inhibitor. Since neither calcein-AM nor CsA are fully specific to Pgp on their own, we also generated ABCB1 knock-out iPSC line as a Pgp negative control (PTL-SBAD3-ABCB1-KO). SBADA3 iPSC were transfected with two plasmid DNAs: one expressing Cas9 together with green fluorescent reporter (GFP) and the other expressing gRNAs (Fig. 7A). PCR and fragment analysis identified multiple homozygous clones with frameshift deletion mutation from a total of 400 + single cell clones screened (Fig. 7A). Next generation sequencing through amplicon sequencing confirmed 116 base pairs frameshift deletion in identified homozygous clones D1, D2, B6 & B12. Clone D1 was differentiated into PTL. We did not observe any differences in differentiating the SBAD3-ABCB1-KO line compared to the wild type line (DLL1 and megalin protein expression are shown in Supplementary Figure S4). The calcein-AM assay showed that in ABCB1-KO PTL cells, higher fluorescence levels of calcein were detected inside the cells, compared to cells expressing ABCB1 (Fig. 7B). CsA had no effect on calcein accumulation in PTL derived from SBAD3-ABCB1-KO clone D1, demonstrating the specificity of the combination of CsA and calcein-AM for Pgp. All other cell types showed a marked increase in calcein accumulation in the presence of CsA (Fig. 7B).

### Comparison to human primary proximal tubular cells

**Figure 8:**
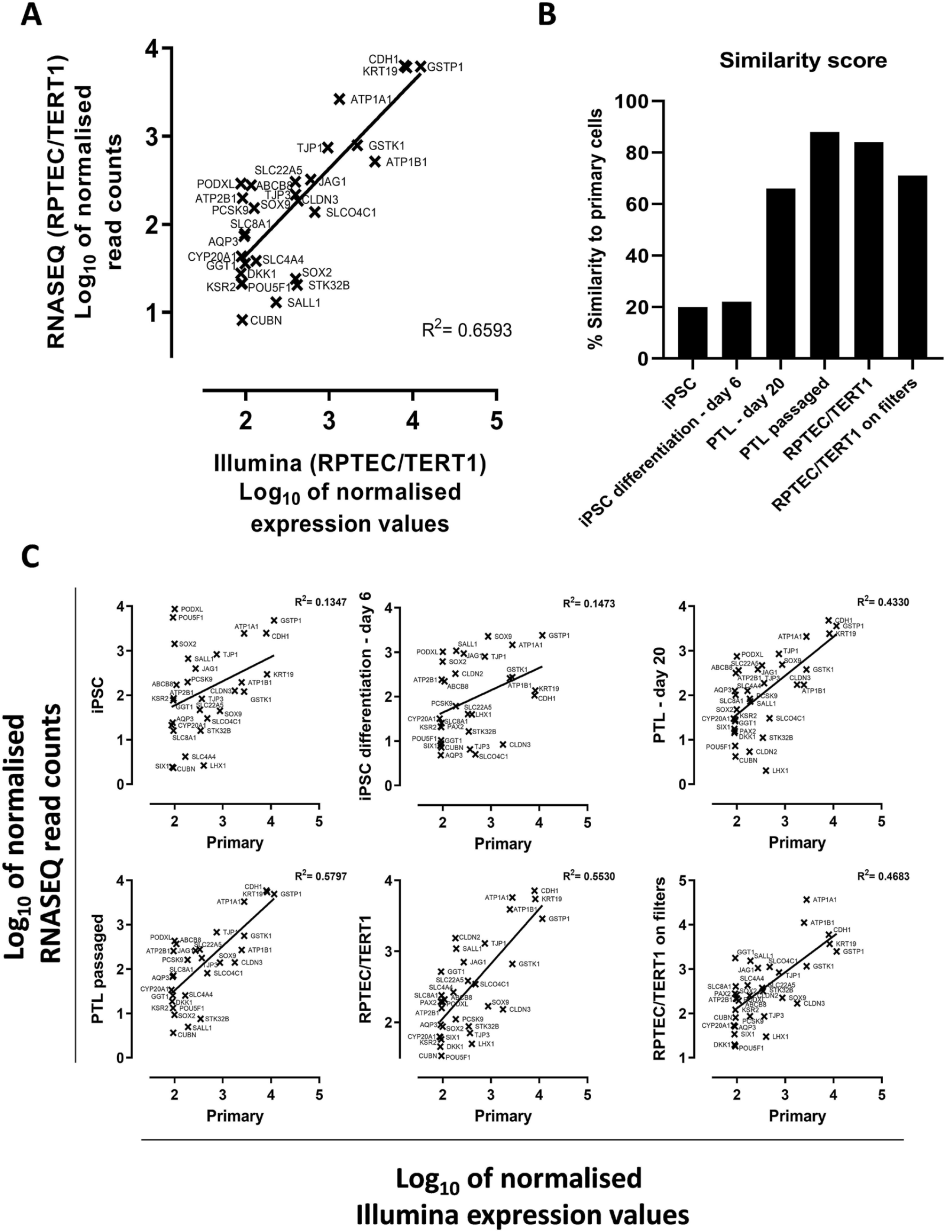
Comparison of RNASeq data to human primary proximal tubular cells (primary). (**A**) Comparison of log10 normalized values of differentiated RPTEC/TERT1 gene expression levels obtained from RNASEQ and Illumina microarray platforms based on list of genes present in Fig. 2B. The genes below the median coefficient of variance were selected for comparison. (**B**) Represents the % similarity of primary cells to each cell type based on normalizing R2 correlation value of figure A (i.e. R2 = 0.6593) to 100% to eliminate platform mismatches. (**C**) Selected genes obtained from panel A are used to compare log10 normalized gene expression data of iPSC, day 6 of iPSC differentiation, PTL-day 20, PTL passaged, RPTEC/TERT1 differentiated on plastic, and RPTEC/TERT1 differentiated on filters obtained from RNSAEQ with primary cells log10 normalized gene expression data obtained from Illumina platform.

Gene expression levels of a subset of genes obtained using the RNASeq experiment (Fig. 2B) was compared to primary human proximal tubular mRNA levels from a Illumina microarray based study^8^. By normalising Pearson R^2^ score of RPTEC/TERT1 from both platforms to 100% (R^2^-0.6593) (Fig. 8A) similarity scores were calculated between human primary proximal tubular cells and iPSC, day 6 of iPSC differentiation, PTL, Passaged PTL, RPTEC/TERT1 grown on plastic and filters (Fig. 8B,C). iPSC and day 6 of iPSC differentiation showed least similarity to primary cells (20% and 22% respectively) whereas PTL and passaged PTL showed higher similarity to primary cells (66% and 88%) (Fig. 8B). The correlation plots of the individual genes are given in Fig. 8C.

## Discussion

In the present study, we describe the differentiation of iPSC into cells with a renal proximal tubule-like phenotype by applying a step-wise exposure of small molecules and growth factors. RNASeq analysis was employed to characterize genome wide gene expression levels at different stages during differentiation. CellNet has been used previously to successfully perform unbiased target cell or tissue type identification by matching RNASeq to reference samples^46^. Using this approach, the Gene Regulatory Network of PTL, clustered with kidney data set, although not to the same extent as RPTEC/TERT1 cells. However, similarity scores to other tissue types was extremely low. It should be noted that the training datasets used in CellNet come with limitations, including the use of single end reads from the RNASeq data and heterogeneity of the datasets^34^. For example, training datasets are restricted to cell and tissue datasets publicly available and are mainly based on tissues rather than single cell types, with the exception of the datasets for ESC and blood cell types.

We compared our data to primary human proximal tubule mRNA from an earlier Illumina chip-based study^8^ from our group. Since these platforms are fundamentally different, we needed to devise a method to align them for this to be scientifically meaningful. We thus used the differentiated RPTEC/TERT1 data that was on both platforms to eliminate non-corresponding gene expressions and to create a meaningful similarity score using a sub-set of genes of highly correlating genes. This was validated in the RPTEC/TERT1 Illumina vs RPTEC/TERT1 sequencing plot (Fig. 8A, R^2^, 0.6593). Correlation and similarity scores of undifferentiated iPSC as well as day 6 of differentiation to primary proximal tubular cells was relatively low, while the correlation and similarities scores of PTL, passaged PTL and RPTEC/TERT1 cells to primary proximal tubular cells was much higher. Thus, the CellNet method and the comparison to primaries concur, with the exception of the passaged PTL, where the CellNet showed a weaker correlation to kidney tissue and the comparison to primary cells showed a stronger correlation. This is possibly due to the fact that the primary cells were also passaged and therefore might be a similar divergence from the original tissue.

The RNASeq data revealed that PTL go through renal developmental stages during the course of differentiation. The kidney derives from the mesoderm, more specifically the intermediate mesoderm (IM), from which the metanephric mesenchyme (MM) and the ureteric bud (UB) arise (reviewed by O’Brien et al.^47^). During the branching process of the UB, a population of mesenchymal cells will arrange into a pre-tubular aggregate, which in turn epithelializes to form a renal vesicle. From each renal vesicle a nephron will develop by going through the stages of comma-shaped and S-shaped- bodies^48^. Our protocol started off by generating IM cells as previously reported by Araoka et al.^49^. By further addition of FGF9 on day 3, cells differentiated further and on day 6 of differentiation, cells expressed high levels of SALL1, DLL1, GREB1 and CDH6, whereas CITED1 and SIX2 levels were absent or low, respectively (Fig. 2B). During the development of this protocol, other growth factors, including FGF2 and LIF were tested on day 3 to drive the generated IM cells into renal cells, but did not work as well as FGF9 (data not shown). Knowledge of mammalian renal development has recently been refined through the availability of single cell sequencing^50,51^, which demonstrates that there is a less binary type expression of specific markers as many overlap several developmental stages. Markers of the cap mesenchyme (CM) include SIX2, CITED1, PAX2/8, EYA1 and SALL1^52^ and it has been suggested that CITED1 is a marker of uninduced CM cells and expression is not prolonged after initial epithelialization^53^. This suggests that by day 6 of our protocol, PTL most likely already passed the CM stage, as no CITED1 expression was detected in the RNASeq. Comparing markers expressed by the PTL on day 6 with known markers of later renal developmental stages, including the renal vesicle or the S-shaped bodies, many overlaps were observed. These include DLL1, SOX9, GREB1, JAG1 and CDH6 which were highly expressed on day 6 as well as PCSK9, DKK1 and BMP2 which were expressed at low levels on day 6. All of these are recognized as markers of the distal part of the renal vesicle^54^ that will give rise to the proximal tubules. CDH6 expression has previously been reported as a marker for proximal tubular progenitor cells^55^ and expression peaked at day 6 of PTL differentiation and reduced again by day 20. Many of these markers, including DLL1 and GREB1 persist during stages of S-shaped bodies, it was therefore difficult to conclude whether the cells have gone beyond renal vesicles. Since we did not utilize single cell sequencing in this study, we cannot exclude the possibility that day 6 represent a mixture of different renal development stages.

In the human body, megalin (LRP2) expression is highest in the proximal tubules and at lower levels in non-renal tissues, including lung alveoli, eye, gall bladder, placenta, parathyroid gland, thyroid gland and ear^33,56^. In the proximal tubule, megalin is involved in uptake of proteins and certain drugs via the megalin/cubilin endocytosis system^33^. Megalin expression first arose at day 3, increased thereafter and was maintained through-out the observed period. In contrast RPTEC/TERT1 cells exhibited no mRNA or protein for megalin. We also demonstrated that PTL can perform albumin uptake, where RPTEC/TERT1 had no such activity (Fig. 6B). The lack of functional megalin in RPTEC/TERT1 cells is contrary to what was reported in the original paper, although in that study there was no positive control included^7^. Here, we included LLC-PK1 as a positive control, which have high levels of albumin uptake. Megalin expression at an mRNA and protein level, taken together with the observation that labelled albumin appeared in dense spots in LLC-PK1 and PTL, suggests that is uptake is indeed megalin mediated endocytosis.

Functional transporters, that are involved in drug handling, are another desired key feature for iPSC-derived toxicity testing models. Due to its wide substrate affinity Pgp (ABCB1) is one of the most studied efflux transporters in ADMET investigation of pharmaceutical compounds. ABCB1 was not detected at an mRNA level in PTL and was extremely low in RPTEC/TERT1 cells. However, as observed for some other transporters, it seems that Pgp protein expression and/or activity is not well represented by mRNA levels^57^. Here utilizing calcein-AM and CsA, we could demonstrate that, despite having low ABCB1 mRNA counts, RPTEC/TERT1 and PTL showed functional Pgp activity. ABCB1 knockout iPSC differentiated into PTL, showed no CsA inhibitable calcein accumulation.

Differentiation status of PTL could be maintained over several days, which is important for toxicity testing, in particular testing that goes beyond 24 h acute exposures, e.g. repeated dose toxicity testing. Long term stability is often a problem with iPSC-derived target cells. Previously reported protocols to directly drive iPSC into proximal tubular like cells did not report long term stability and presence of proximal tubular specific markers were only shown for 24 h after differentiation^32^. Other advantages of this model regarding the application for toxicity testing is that the cell culture medium of PTL is serum free and the use of small molecules, including CHIR99021 and TTNPB, is significantly cheaper and easier to handle (e.g. allows freeze-thawing, less batch to batch variations) compared to using growth factors. Furthermore, passaging of differentiated PTLs offers the opportunity to differentiate in bulk, and re-plate the cells in the desired well-format required and provides the option to freeze the cells in liquid N_2_ and thaw them again when required. For passaging PTL the addition of the TGF-beta inhibitor GW788388 hydrate was essential as the cells tended to dedifferentiate into fibroblast like cells when re-plated. TGF-beta plays a critical role in epithelial to mesenchyme transition (EMT) and addition of TGF-beta to cultured epithelial cells has been previously shown to initiate EMT^58^. In addition, high levels of TGF-beta have been recorded in fibrotic kidney in patients and targeting TGF-beta is currently explored for the treatment of fibrotic kidney diseases^59^. PTL at 10 days post passage, showed comparable responses as unpassaged PTL, to parathyroid hormone, a key hormonal characteristic of the proximal tubule cells in vivo and in vitro^7^, as well as albumin uptake and Pgp efflux. In addition, brush border structures with microvilli were maintained well in passaged PTL.

While PTL showed several important and specific characteristics of renal proximal tubular cells, including functional hormone response to parathyroid hormone, Pgp efflux, megalin facilitated endocytosis and formation of brush border structures, we are aware that they had also limitations. Like most iPSC-derived cell models, including hepatocyte-like cells^60^ and cardiomyocyte-like cells^61^, PTL may have not been entirely matured as compared to cells coming from adult human kidneys such as primary human proximal tubule cells or RPTEC/TERT1 cells and they were not expressing high levels of all desired proximal-tubular specific genes. Although we could not at this stage rule out the possibility that the differentiated PTL represented a fully pure population we have not identified sub-populations. Expression of markers characteristic for podocytes, loop of Henle cells, distal tubular cells, collecting duct cells or endothelial cells were mostly absent or expressed at low levels (Figs. 2B and 3C). mRNA expression for SLC8A1, ENOX1, EGF, CLIC5 and PLA2R were expressed at low levels in PTL and with the exception of EGF were also present in RPTEC/TERT1 cells. However, this is not necessarily evidence of non-proximal tubule phenotypes as these markers have also been found at low levels in S3-type proximal tubular cells^35^. AQP3 expression was found in PTL and RPTEC/TERT1 cells and had previously been reported in cultured human primary proximal tubular cells and in the human renal proximal tubular cell line HK2 and that may be due to induction by transferrin that is present in the cell culture medium^62^. On the other hand, KRT19 (cytokeratin 19), which is preferentially expressed in the distal nephron, exhibited relatively high mRNA levels in PTL, although this was also true for RPTEC/TERT1 cells.

In conclusion, we report a differentiation protocol, using a combination of small molecules and growth factors, to drive iPSC into cells with a renal proximal tubular-like phenotype within 14 days. PTL cells expressed proximal tubular specific genes and proteins, including megalin (LRP2), PAX2 and PTH1R and demonstrated proximal tubule like hormonal responses, megalin-facilitated endocytosis and Pgp efflux transport. PTL could be maintained for a minimum of 7 days and passaged in the presence of the TGF-beta inhibitor GW788388 hydrate. To the best of our knowledge this is the first time to report an iPSC-derived proximal tubular cell model that shows stability over an extended time frame and as such this protocol will be useful for generating iPSC derived PTL cells for molecular and in vitro toxicological investigations. Future work will be required however, to further improve the phenotype for example by upgrading the microenvironment via the use of microporous supports and microfluidics.

## Materials and methods

### iPSC culture

iPSCs were obtained from the StemBANCC consortium^63^ and are now available from the European Bank for induced Pluripotent stem cells (EBiSC) (https://cells.ebisc.org/). Cells have been generated by non-integrative Sendai virus transfection, using the CytoTune-ips 2.0 reprogramming kit as previously described^64^. iPSCs were routinely cultured on Geltrex coated plates (Life Technologies A1413302), in mTeSR1 medium (StemCell Technologies 05850) and passaged with EDTA (0.02% Versene, Lonza BE17-711E) two times per week. The following three iPSC lines were used: SBAD2-clone 1 (SBAD2), SBAD3-clone 1 (SBAD3) and SFC086-03-01 (SFC086).

### RPTEC/TERT1 cell culture conditions

RPTEC/TERT1 cells were cultured in proximal tubular cell culture medium (medium 2), consisting of 1:1 mixture of DMEM:F12, 2 mM glutamax 5 μg/ml insulin, 5 μg/ml transferrin and 5 ng/ml sodium selenite, 10 ng/ml epithelial growth factor (Sigma Aldrich E9644) and 36 ng/ml hydrocortisone (Sigma-Aldrich H0135) as described previously^8^, with the addition of 100 U/ml penicillin, 100 μg/ml streptomycin and 0.5% FCS. Cells were matured after culturing them for a minimum of 10 days at confluence as previously described^8^.

### Podocytes, HepG2 and LLC-PK1 culture

iPSC SBAD2 were differentiated into renal podocytes as reported previously^31^. HepG2, LLC-PK1 and LLC-PK1 transfected with UMOD (LLC-PK1-UMOD)^65^ cells were cultured in medium containing 1:1 mixture of DMEM: F12 (Gibco 11966-025 and Gibco 21765-029), 2 mM glutamax (ThermoFisher Scientific 35,050,038), and 100 U/ml penicillin, 100 μg/ml streptomycin with either 7% FBS (LLC-PK1, LLC-PK1-UMOD) or 10% FBS (HepG2).

## Differentiation into proximal tubular -like cells (PTL)

### ECM preparation

RPTEC/TERT1 cells were cultured on the specified multi-well plates until four days after reaching confluence. Wells were washed with sterile ddH_2_O once and incubation with 35 mM NH_3_ solution for 5 min (as previously described by Lu et al.^66^). Wells were washed 3 times with PBS to remove cellular debris. The wells containing the RPTEC/TERT1 ECM coat were used either immediately or stored at 4 °C for up to 4 weeks.

### Differentiation into PTL

The differentiation is based on a multi-step protocol to induce several stages during renal development (Fig. 1). The first step was a modified protocol by Araoka et al.^49^ to induce intermediate mesoderm (IM) using the small molecules CHIR99021 (abcam 120190) and TTNPB (Sigma T3757). In the next step, the growth factor FGF9 was added (ThermoFisher Scientific PHG0194) and the third and final step, hydrocortisone and EGF were added to the medium as previously described^8^. In more detail, iPSCs were washed in PBS (ThermoFisher Scientific 14190-094) and detached from the culture plate by incubating with accutase for 3–5 min. The cells were then seeded at a concentration of 4 × 10^4^/cm^2^ on either Geltrex-coated plates or ECM-coated plates (specified in corresponding figure legend or methods section) in differentiation medium (medium 1), consisting of 1:1 mixture of DMEM:F12 (Gibco 11966-025 and Gibco 21765-029), 2 mM glutamax (ThermoFisher Scientific 35050038), 5 μg/ml insulin, 5 μg/ml transferrin and 5 ng/ml sodium selenite (Sigma Aldrich I1884), supplemented with 3 µM CHIR99021, 1 µM TTNPB and 10 µM Rock inhibitor (abcam 120129) (day 0). After 42 h (day 2) cells were incubated with medium 1 supplemented with 1 µM TTNPB. After 72 h (day 3) cells were incubated in proximal tubule cell culture medium (medium 2, as described above), supplemented with 10 ng/ml FGF9. After a minimum of 120 h (day 5) to a maximum of 140 h (day 6), cells were fed with medium 2 without any additional supplements. Cells were then fed every two to three days. From day 14 onwards, cells were referred to as PTL. From a 10 cm dish (55 cm^2^) an average of 1.6 × 10^7^ ± 0.32 × 10^7^ cells were obtained. PTL were employed in experiments directly or after they have been passaged between day 14 and day 20.

### Passaging and freezing of differentiated PTL

For subsequent assays, PTL were either employed directly or passaged into another well format on day 16 (specified in each figure legend). For passaging, cells were washed once in PBS, followed by washing once in 0.02% EDTA. Cells were then incubated with TrypLE (ThermoFisher Scientific 12604–013) for 5 min at 37 °C and collected in 10 ml PBS containing 10% FSC (ThermoFisher Scientific 10270-106). Cells were centrifuged at 300 g at 4 °C and resuspended in 1 mL PBS containing DNAse (20 Kunitz units/ ml). After incubation of 5 min, cells were centrifuged again for 5 min at 4 °C and pellets were resuspended in medium 2 supplemented with 1 µM GW788388 hydrate (Sigma Aldrich SML0116), 2% FCS and 10 µM Rock inhibitor. Cells were plated out at a splitting ratio between 1:3 and 1:6 without additional coating unless otherwise specified. Cell count was regularly conducted using the Trypan Blue method using a 1:1 ratio of cell suspensions and 0.4% trypan Blue to determine the seeding densities of living cells. Cell counting was performed using the Luna II (Logo Biosystems) automated cell counter. Optimal cell seeding numbers ranged between 2.5 × 10^6^ and 5 × 10^6^ cells per 96-well plate (30.72 cm^2^). The following day after plating, cells were fed with medium 2 supplemented with 1 µM GW788388 hydrate without FCS or Rock inhibitor. From then onwards, cells were fed every 2 to 3 days with proximal tubular medium (medium 2) supplemented with 1 µM GW788388 hydrate. Passaged PTL continued to proliferate and usually reached confluence within 2 to 4 days, depending on seeding density. Passaged PTL were utilized between day 4 and day 15.

### RNA isolation and RNA sequencing

iPSCs (line SBAD3) were differentiated in 6-well plates (n = 4 replicates of a single differentiation) and harvested at the following time points: day 0 (undifferentiated iPSC), day 6, and day 20. The cells that were harvested at day 20 were grown for 4 days in the presence of 1 µM GW788388 hydrate (day 16-day 20). In addition, cells were passaged on day 16 and re-plated in 6-well plates. These were harvested on day 4 after plating. For comparison, RPTEC/TERT1 cells grown on plastic or transwells (PET filter, 1 µm pore size-Millipore: MCRP24H48) were harvested after contact inhibition and differentiation^8^. Prior to lysis, cells were washed in PBS twice, followed by incubation in 500 µl TRIzol. Cells were transferred to an Eppendorf tube and samples were vortexed and incubated for 5 min at RT. Lysates were then stored at − 80 °C until RNA isolation. After resuspension in TRIzol RNA was extracted using the Qiagen RNAse-Free Mini Kit (217004) and possible remaining DNA was removed with DNase digestion with the Qiagen RNase-Free DNase Set (79254). After assessment of RNA quantity and quality using Qubit RNA HS Assay Kit (Q32855) and Agilent RNA 6000 Nano Kit (5067-1511), sequencing libraries were prepared using the Lexogen SENSE mRNASeq Library Prep Kit V2 (001.96). Finally, the libraries were sequenced on Illumina HiSeq2000 in 100 bp paired-end (at an average of 12 million reads per samples). The obtained fastq files were trimmed using Trimmomatic (version 0.33)^67^ to remove the bad quality sequences and remaining reads were mapped to the human genome (Ensembl version 84) using Bowtie2^68^ (v. 2.2.6) and genes were quantified with RSEM^69^ (v.1.2.28). The gene expression was normalized using DESeq2^70^ and the outliers were removed. While normalizing the RNASeq data, one sample “passaged PTL_R3” was removed due to poor coverage. CellNet^46^ was used for assessing the cell types during the development of the iPSC to PTL. For the CellNet analysis all genes that were expressed by each cell type were taken into account.

The color scheme for heatmap was generated either with overall color scheme with min and max values set based on the entire datasets (Fig. 2A) or using relative color scheme with min and max values taken from each row (Fig. 2B).

### Immunofluorescence

iPSCs (SBAD2, SBAD3 and SFC086) were differentiated into PTL for 16 days on Geltrex and RPTEC/TERT1 were cultured for at least 15 days in a CellCarrier ultra-black 96 well plates. Cells were fixed with 4% PFA for 20 min and permeabilized with 0.1% Triton X-100 for 10 min. Samples were blocked with 2% BSA for 1 h. Samples were then incubated with the following primary antibodies for 2 h at RT: Oct3/4, WT1, PAX2, megalin, ZO3, ACE2, HNF4A, UMOD, Nephrin and followed by Alexa Flour 488 or 546 secondary antibodies for 1 h at RT along with phalloidin and Hoechst 33342. Catalogue numbers and dilutions of antibodies are presented in Supplementary Table S2. Samples were imaged using the confocal Operetta CLS high content imager (Perkin Elmer) with 40X or 63X water objective, Numerical Aperture: 1.1 or 1.15 and the images were collected using Harmony software 4.8.

### Western blotting

iPSCs (SBAD2, SBAD3 and SFC086) were differentiated into PTL in 6-wells for 16 days. Cells were then passaged on day 16 and cultured for an additional 2 weeks. At various time points, cells were harvested by washing them in PBS and scraping them in RIPA buffer (Sigma, 0278) containing 1% protease inhibitor cocktail (Sigma P8340) on ice. Protein fractions were centrifuged at 8000 g and insoluble pellets were discarded. Lysates were stored at -20 °C prior to use. Western blotting was carried out using the NuPAGE gradient gels (ThermoFisher Scientific), as previously described^71^ and 30 µg of protein was loaded into each well. For small and medium size proteins 4–12% gradient Bis–Tris gels were used with MOPS buffer (NP0001). For megalin 3–8% gradient Tris–acetate gels (EA0375) and Tris acetate buffer (cat. LA0041) was used. Proteins were transferred onto low PVDV membranes (Millipore IPVH00010) using NuPAGE Transfer buffer (NP006) in the presence of 10% methanol for MOPS gels or without an addition of methanol for Tris–acetate gels. Membranes were blocked in 5% skimmed milk for 1 h at RT or overnight at 4 °C. The following primary antibody were incubated for either 2 h at RT or overnight at 4 °C: rabbit anti-LRP2/megalin, rabbit ant-DLL1 antibody, mouse anti-OCT3/4 antibody and rabbit anti-tubulin. After three washes in PBS-T, secondary antibody was incubated for 1 h at RT using either anti-rabbit HRP or anti-mouse-HRP at 1:10,000. After three washes in PBS-T ECL substrate (32106, Pierce) was added for 5 min and membranes were imaged on a LAS4000 western blot imager (GE Healthcare).

### Scanning electron microscopy (SEM)

PTL were differentiated on 24-well transwell inserts (PET filter, 1 µm pore size-Millipore: MCRP24H48) coated with Geltrex. Cells were either fixed on day 16 (unpassaged samples) or passaged on day 16 and cultured for an additional 10 days on 24-well transwell inserts. RPTEC/TERT1 cells were cultured on 24-well transwell filters for comparison. Cells were fixed in a pre-warmed fixative (4% paraformaldehyde (PFA), 5% glutaraldehyde (GA) in 0.1 M phosphate buffer (PB)) that was added gently to equal volume of cell culture medium to the cells, followed by incubation in a second fixation solution (2% PFA, 5% GA in 0.1 M PB) for 1 h at RT. Samples were stored at 4 °C until they were further processed for SEM. Prior to SEM, the samples were dehydrated using an ethanol series. To reduce sample surface tension, samples were immersed in hexamethyldisilizane (Sigma-Aldrich) for 30 min and left to air dry. Afterwards, samples were mounted on aluminium SEM stubs and sputter-coated with a 4 nm platinum-palladium layer using a Leica EM ACE600 sputter coater (Leica Microsystems, Illinois, USA). Images were acquired at 3 kV using a Zeiss Sigma 300 SEM (Zeiss, Germany) located at the Cellular Imaging core facility of the Amsterdam UMC (Universitair Medische Centra) in Amsterdam, the Netherlands.

### PTH response

To determine hormonally stimulated cAMP responses three lines of iPSCs (SBAD2, SBAD3 and SFC-086) were differentiated on ECM coated 24-well plates (Greiner) for 16 days. PTL were used either on day 16 (unpassaged) or passaged on day 16 and re-plated on uncoated 24-well plates (passaged). Passaged cells were cultured for another 11 days to allow them to grow back to confluence and to allow monolayer maturation. Prior to stimulation, cells were incubated in 100 µM of the cAMP phosphodiesterase inhibitor 3-Isobutyl-1-methylxanthine (IBMX; Sigma I5879) for 1 h. Parathyroid hormone fragment 1–34 human (10^–7^ M; Sigma P3796) and [Arg8]-Vasopressin (100 mU/ml; Sigma V9879) were applied to the cells for 20 min. Cells were then lysed in 0.1 M HCl, and lysates were stored at − 20 °C. Total cAMP concentration was determined using the Direct cAMP ELISA kit (ADI-900-163, Enzo Life Sciences).

### Albumin uptake

PTL, passaged PTL, RPTEC/TERT1 (negative control) and LLC-PK1 (positive control) cells were cultured in CellCarrier ultra-black 96 well plates. Cells were washed with medium 2 before adding BSA-Alexa 647. Cells were incubated with BSA-Alexa 647 for every 1 h until 6 h. After 6 h the BSA-Alexa 647 was aspirated, and Hoechst 33342 (0.5μg/ml) was added to the cells and incubated for 15 min. After the incubation, samples were imaged using confocal Operetta CLS high content imager (Perkin Elmer) using 63 × water objective, with the brightfield channel (white light) and 2 fluorescence channels: Ex615-654 nm Em655-760 nm (BSA-alexa647) and Ex355-385 nm Em430-500 (Hoechst 33342). The images were analyzed with the software Harmony 4.8 (Perkin Elmer). The uptake of BSA-alexa647 is visible as fluorescent spots in the cells. The spots were selected, and the total fluorescence intensity of the spots was determined. The cell count per well was performed with the Hoechst staining and the normalization was done, by expressing the sum of the fluorescence intensity of BSA-Alexa647 in all the spots per cell. The experiment is performed in triplicate.

### Generation of ABCB1 KO iPSC using CRISPR/Cas9 gene editing

To construct pEF1-Cas9-T2A-GFP plasmid vector for expression of Cas9 and GFP proteins, Cas9 sequence was synthesized by ThermoFisher Scientific. The Splice acceptor-T2A-GFP was amplified by PCR from a plasmid using primer pair 5´ACGCGTCACTCTCGAGGGAGAGGGCAGAGGAAGTCTTCTAAC3´ and 5´-ACGCGTCACTCTCGAGCCATAGAGCCCACCGCATCCCC-3´. The gRNAs were designed using AstraZeneca’s CRISPR3 tool and then cloned in pMlu vector under hU6 promoter. The guide RNA sequences are gRNA1: TTTATAGTAGGATTTACACGTGG and gRNA2: ACCAATTCCTTCATTAATCTTGG.

For gene editing, cells were cultured using the Cellartis DEF-CS 500 culture media system (Clontech-Takara, Y30017). The wild type SBAD3- clone 1 iPSC cell line was used for generation of ABCB1 KO lines. SBAD3-clone 1 cells were harvested at passage P21 using TrypleE (Gibco); 300,000 cells/well of a 12 well plate were transfected with pEF1-Cas9-T2A-GFP and pMlu-hU6-gRNA plasmid vector using Lipofectamine LTX (ThermoFisher Scientific). After 48 h of transfection, based on GFP expression cells were FACS sorted as single cell based on GFP expression using Fortessa (BD Biosciences) and single cell cloning was performed in 384 well plates to identify pure clones. Approximately 400 single cell clones were analyzed using PCR (Polymerase Chain Reaction), fragment analysis and next generation sequencing through amplicon sequencing methods. For PCR & Fragment Analysis, DNA from clones were isolated using Modified Gitschier Buffer (MGB) and PCR was performed using Phusion high fidelity Taq pol (ThermoFisher Scientific). Fragment analysis of PCR products of ABCB1 clones were performed using 5200 Fragment Analyzer (Agilent). Sequence analysis and the integrity of the mutation was confirmed with next generation sequencing analysis through amplicon sequencing using Illumina NextSeq500 (Illumina).

### Calcein-AM assay

iPSCs (SBAD2, SBAD3 and SFC086) were differentiated into PTL for 16 days in a CellCarrier ultra-black 96 well plates coated with Geltrex. Cells were either used in transport studies on day 16 (unpassaged samples) or passaged on day 16 and cultured for an additional 10 days. As a negative control, PTL derived from iPSC SBAD3-ABCB1-KO clone D1 cells were used. RPTEC/TERT1 cells were let to differentiate in the same plates for at least 15 days. For the calcein accumulation assay cells were incubated in phenol red free medium for 1 h with and without CsA (5 μM, 239835, Sigma-Aldrich). After incubation cells were stained with Hoechst 33342 and a medium containing calcein-AM with the same CsA conditions was applied. Samples were imaged using the Operetta CLS high content imager with 63X water objective in confocal mode. Images were collected and analyzed using Harmony software 4.8. Analysis was performed by calculating the whole image fluorescence sum per sample divided by the number of cells.

### Statistical analysis

Data is represented as mean ± standard deviation (SD) and statistical analysis was performed with one-way ANOVA by Dunnett’s multiple comparison test. For multiple group kinetics comparison, statistical analysis was performed with two tailed unpaired Student’s t-test of the area under the curve (AUC) Analysis was done using the GraphPad Prism and *P*-value < 0.05 were shown to be statistically significant.

### Comparison of gene expression data sets from Illumina and RNASEQ

RPTEC/TERT1 and human primary proximal tubular cells gene expression data obtained from Illumina microarray-based transcriptomics^8^ were compared to RNASEQ gene expression data of iPSC, day 6 of iPSC differentiation, PTL, passaged PTL, RPTEC/TERT1 and RPTEC/TERT1 grown on filters using log transformed DESeq2 normalised values. Gene expression data from the two platforms was compared to the subset of genes listed in Fig. 2B from the same sample type (RPTEC/TERT1 differentiated on plastic). Non-correlating (CV less than 0.179) and non-expressed genes (expression 0) were removed. There were 33 well correlating genes remaining (Pearson R^2^ 0.6893, Fig. 8A). Since this was the same sample type in both platforms this R^2^ represents a 100% similarity and was set as such. This was then used to compare all samples to the expression of the genes in human primary proximal tubule cells.

## Supplementary Information

Supplementary Information.

## Data Availability

The datasets generated during and/or analyzed during the current study are available from the corresponding author on reasonable request. RNASeq data is available at the European Nucleotide archive (ENA) at the EMBL-EBI under the accession numbers PRJEB29471/ERP111777 (datasets named DF, DP and UP refer to differentiated filter, differentiated plastic and undifferentiated proliferating RPTEC/TERT1 cells, respectively) and PRJEB23622/ERP105384.
