# Supplementary material for: Generation and characterization of iPSC-derived renal proximal tubule-like cells with extended stability
Source: Sci Rep. 2021 Jun 2;11:11575. doi: 10.1038/s41598-021-89550-4 (PMC8172841; doi:10.1038/s41598-021-89550-4)
Supplement: Supplementary file 1 — Supplementary Information. [file 41598_2021_89550_MOESM1_ESM.pdf]

Supplementary material

Generation and characterization of iPSC-derived renal proximal tubule-like cells with extended stability

Vidya Chandrasekaran[1], Giada Carta[1], Daniel da Costa Pereira[1], Rajinder Gupta[2], Cormac Murphy[1] Elisabeth Feifel[3], Georg Kern[3], Judith Lechner[3], Anna Lina Cavallo[4], Shailesh Gupta[4], Florian Caiment[2], Jos CS Kleinjans[2], Gerhard Gstraunthaler[3], Paul Jennings[1*] and Anja Wilmes[1*]

[1]Division of Molecular and Computational Toxicology, Department of Chemistry and Pharmaceutical Sciences, Vrije Universiteit Amsterdam, Amsterdam, The Netherlands.

[2]Department of Toxicogenomics, Maastricht University, School of Oncology and Developmental Biology (GROW), Maastricht, The Netherlands.

[3]Institute of Physiology and Medical Physics, Medical University of Innsbruck, Innsbruck, Austria.

[4]Discovery Sciences R&D, AstraZeneca, Gothenburg, Sweden

**Corresponding Authors*

Supplementary Table S1

Name	Description	Purpose	Reference
**iPSC – induced pluripotent stem cells**	Undifferentiated induced pluripotent stem cells	Used to generate PTL, to create ABCB1 knock out and as a reference time point (day) for PTL differentiation.	(Wilmes et al. 2017)
**PTL (day 6) – proximal tubule like cells**	Immature stage of proximal tubular-like cells. Renal developmental stage: Renal vesicular stage to S-shaped body stage	Required for later stage differentiation and for characterisation of the differentiation process.	
**PTL (day 16 and day 20), also called PTL unpassaged**	Differentiated stage of proximal tubular-like cells, showing a proximal tubular phenotype	Matured PTL – the most advanced stage of our protocol.	
**PTL passaged**	Differentiated proximal tubular-like cells that were passaged (i.e. trypsinised) once (P1).	It is sometimes necessary for certain applications to seed the cells specifically. The ability to passage the cells with a relatively similar characterisation is a big advantage. PTL can be passaged from day 14 onwards. They do, however, need a minimum of seven days to restabilise before use.	
**Podocytes**	iPSC derived podocytes	Use here as a positive control for nephrin expression.	(Murphy et al. 2019)
**SBAD03-ABCB1-KO**	CRISPR/Cas9 generated ABCB1 (P-glycoprotein) knockout.	Used to check the p-glycoprotein specificity of the calcein AM extrusion assay.	
**RPTEC/TERT1 – renal proximal tubular endothelial cell line**	hTERT immortalised human renal proximal tubular epithelial cells from Evercyte GmbH	Cells are a commonly used as a model of the human proximal tubule, especially in toxicological applications.	(Wieser et al. 2008)

**LLC-PK1 (Lilly Laboratories Cell-Porcine Kidney 1)**	Wild type LLC-PK1 cell line	Unlike many proximal tubular cell lines and primary cells in culture the LLC-PK1 expresses a functional megalin mediated endocytosis system.	(Nielsen et al. 1998)
**LLC-PK1-UMOD**	LLC-PK1 cell line transfected with human uromodulin (aka Tamm-Horsfall protein)	These cells were stably transfected with a plasmid containing the wild type human uromodulin gene. Uromodulin is expressed in the thick ascending limb. Cells were used as positive control for uromodulin expression.	(Jennings et al. 2007)
**HepG2**	Human liver cancer cell line	Commonly used cell in molecular biology and toxicology experiments.	(van der Stel et al. 2020)

Supplementary Table S2

Antibody/dye	Company	Catalogue number	Concentration/ Dilution	Application
**ACE2**	R&D systems	AF933-SP	1:250	IF (immunofluorescence)
**Alpha acetylated tubulin**	Invitrogen	32-2700	1:200	IF
**Anti - Goat IgG - Alexa 546**	Invitrogen	A-11056	1:1000	IF
**Anti - Mouse IgG - Alexa 488**	Invitrogen	A-21202	1:1000	IF
**Anti - Mouse IgG - HRP**	Invitrogen	31430	1:10000	WB (western blot)
**Anti - Rabbit IgG - Alexa 546**	Invitrogen	A10040	1:1000	IF
**Anti - Rabbit IgG - AlexaFluor 488**	Invitrogen	A-21206	1:1000	IF
**Anti - Rabbit IgG - HRP**	Invitrogen	31460	1:10000	WB
**BSA – Alexa Fluor 647**	Invitrogen	A34785	10 µg/ml	Albumin uptake assay (Albumin as a substrate for Megalin)
**Calcein-AM**	Sigma-Aldrich	239835	5 µM	Calcein-AM efflux assay (Calcein-AM as a substrate for p-GP)
**DLL1**	Abcam	ab84620	1:1000	WB
**HNF4A**	Sigma	HPA004712	1:50	IF
**Hoechst 33342**	Invitrogen	H3570	1 µg/ml	IF (nuclear staining)
**Megalin**	R&D systems	MAB9578	1:100	IF
**Megalin**	Abcam	ab76969	1:1000	WB
**Nephrin**	Abcam	ab183099	1:100	IF
**Occludin – Alexa Fluor 594**	Invitrogen	331594	1:250	IF
**Oct 3/4**	Santacruz	SC-5279	1:100/1:1000	IF/WB
**PAX2**	Abcam	ab79389	1:100	IF
**Phalloidin-Alexa Fluor 488**	Invitrogen	R37110	1 drop/ml	IF (F-actin staining)
**Tubulin**	Abcam	ab4074	1:1000	WB
**UMOD**	Cedarlane	CL1032A	1:500	IF
**WT1**	R&D systems	AF5729	1:100	IF
**ZO3**	Invitrogen	36-4000	1:250	IF

Supplementary Figure legends

Figure S1: Immunofluorescence imaging showing the phenotype of proximal tubular-like cells SBAD3 and SFC-086 were differentiated into PTL on Geltrex and cells were fixed on day 0, day 6 and day 16 of differentiation and stained with antibodies for selected markers. Images were taken using confocal microscopy with 40X or 63X water objectives. For all the images, scale bar is represented as 20 µm.

Figure S2: Ultrastructural characteristics of proximal tubular-like cells. iPSC (SBAD2, SFC-086) were differentiated into PTL on Geltrex on 24 well transwell filters for 16 days or passaged onto transwell filters from 6 well and grown for 10 days. Samples were processed for SEM images.

Figure S3. Full blot images of western blots shown in Figure 4. (A) SBAD2 line (B) SBAD3 line and (C) SFC-086 line were differentiated on ECM and lysed at several time points. In addition, cells were passaged and lysed at additional time points. Regions shown in Figure 4 are indicated by the black box.Note: Prior to Oct3/4 antibody staining in the passaged SBAD3 (B), another antibody, detecting a 110 kDa protein, was used beforehand. This is indicated with an arrow. One of the membranes (passaged SBAD3, megalin staining, B) had been cut and put together for presenting the full blot.

Figure S4. A. Full gel of PCR as shown in Figure 7. Agarose gel of PCR ABC1B1 products from selected clones. D1, D2, B6 and B12 exhibit a ABCB1 116bp deletion. **B. Differentiation markers for ABCB1-KO cells.** SBAD3-PTL (wild type) was compared to SBAD3-ABCB1-KO at an undifferentiated state (SBAD3-ABCB1-KO-iPSC) and a differentiated stage (SBAD3-ABCB1-KO-PTL) using western blotting (shown in red box). Antibody staining was performed for the markers megalin, DLL1 and Oct3/4.

Figure S1

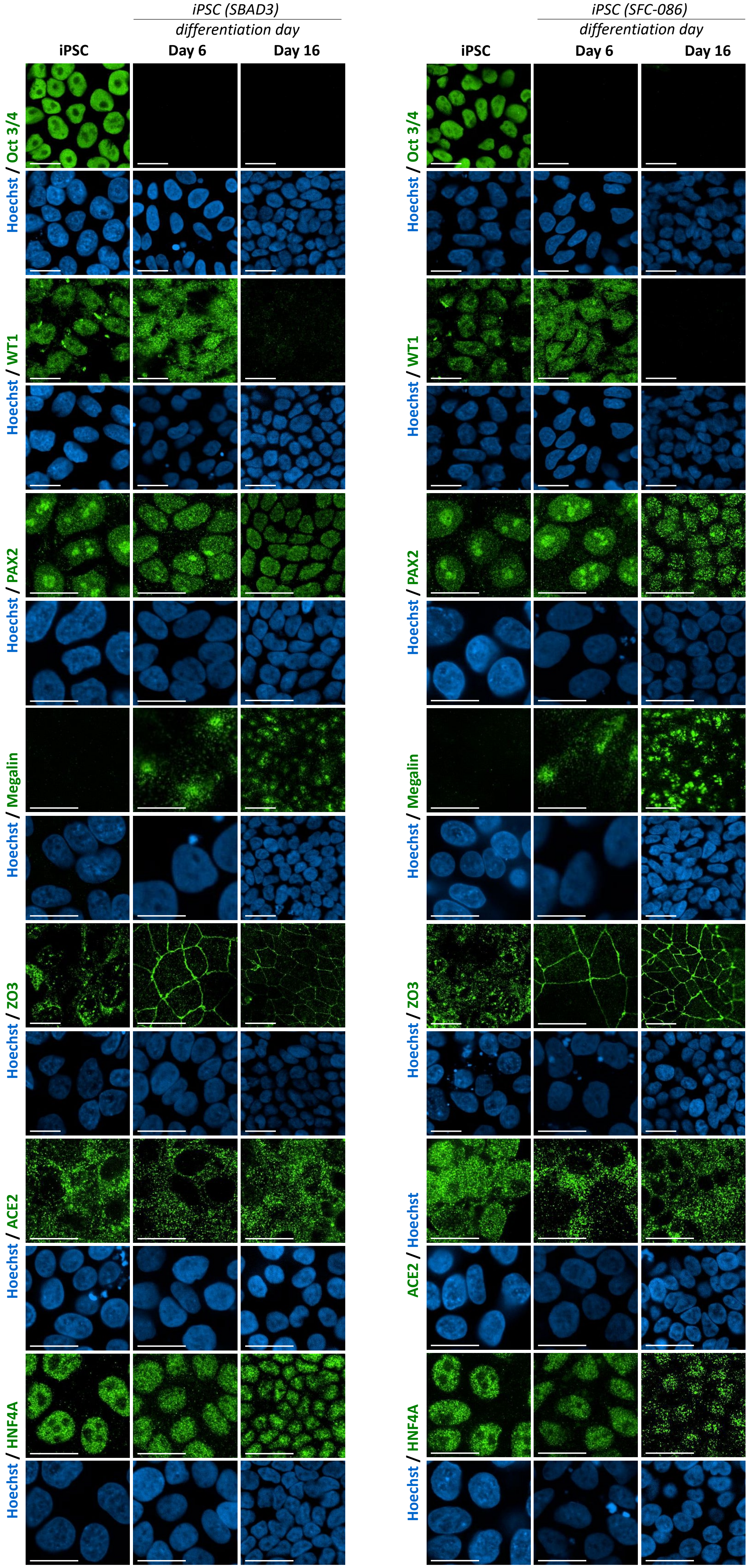
iPSC (SBAD3)
differentiation day
iPSC
Day 6
Day 16
iPSC (SFC-086)
differentiation day
iPSC
Day 6
Day 16
Hoechst / Oct 3/4
Hoechst / WT1
Hoechst / PAX2
Hoechst / Megalin
Hoechst / ZO3
Hoechst / ACE2
ACE2 / Hoechst
Hoechst / HNF4A

Figure S2

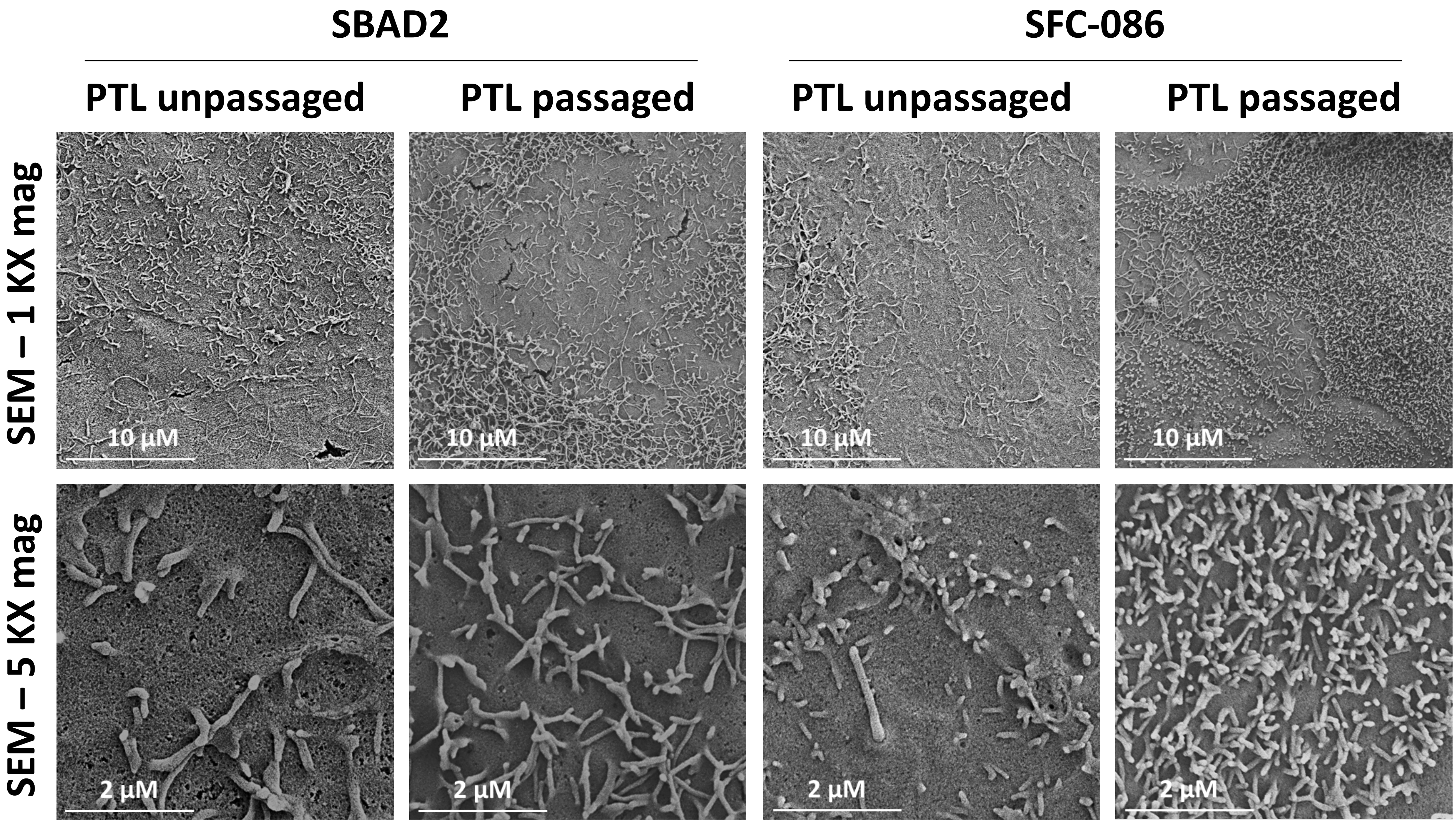
SBAD2
SFC-086
PTL unpassaged
PTL passaged
PTL unpassaged
PTL passaged
SEM – 1 KX mag
SEM – 5 KX mag
10 µM
10 µM
10 µM
10 µM
2 µM
2 µM
2 µM
2 µM

Figure S3A

SBAD2

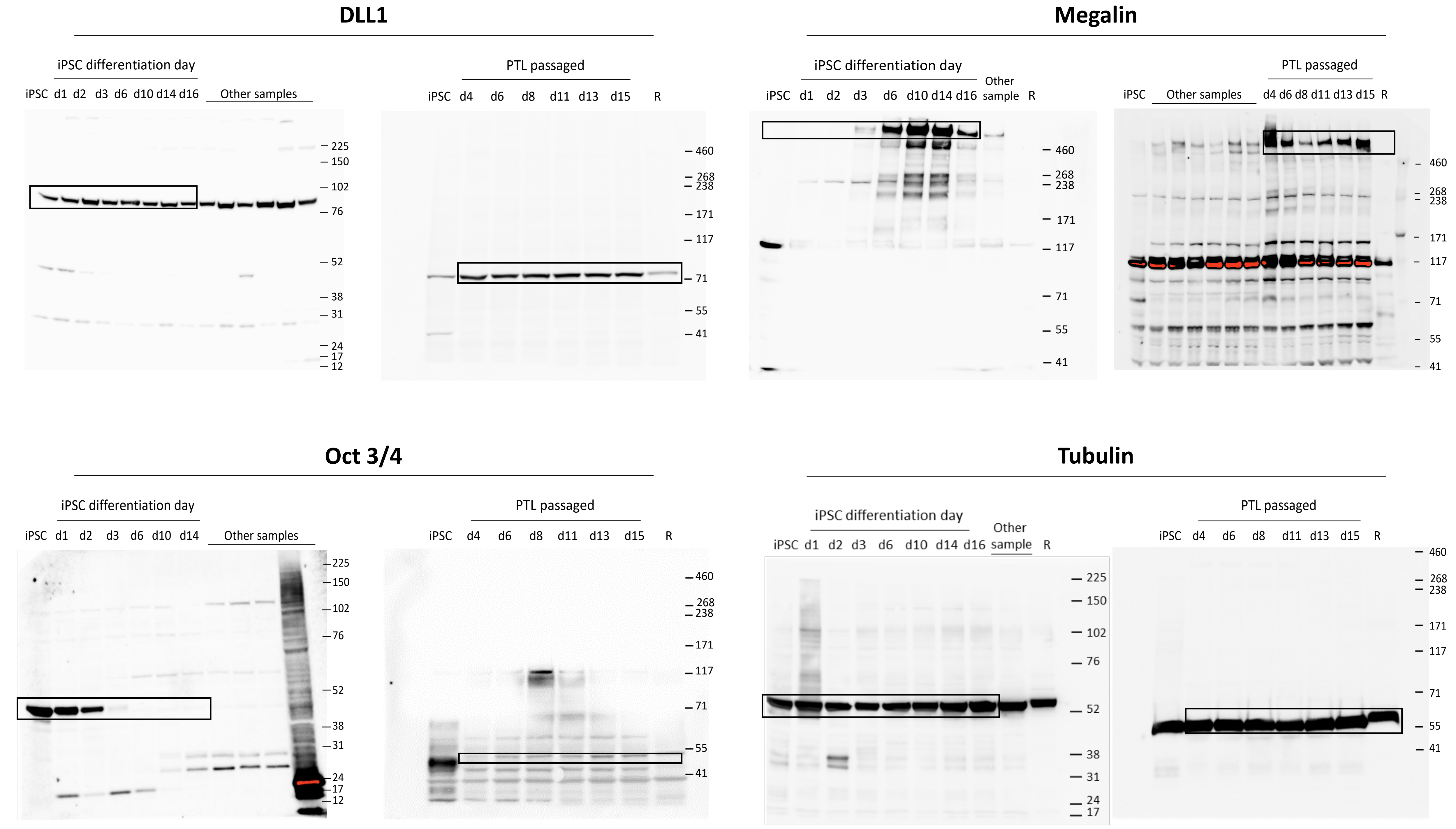

Figure S3B

SBAD3

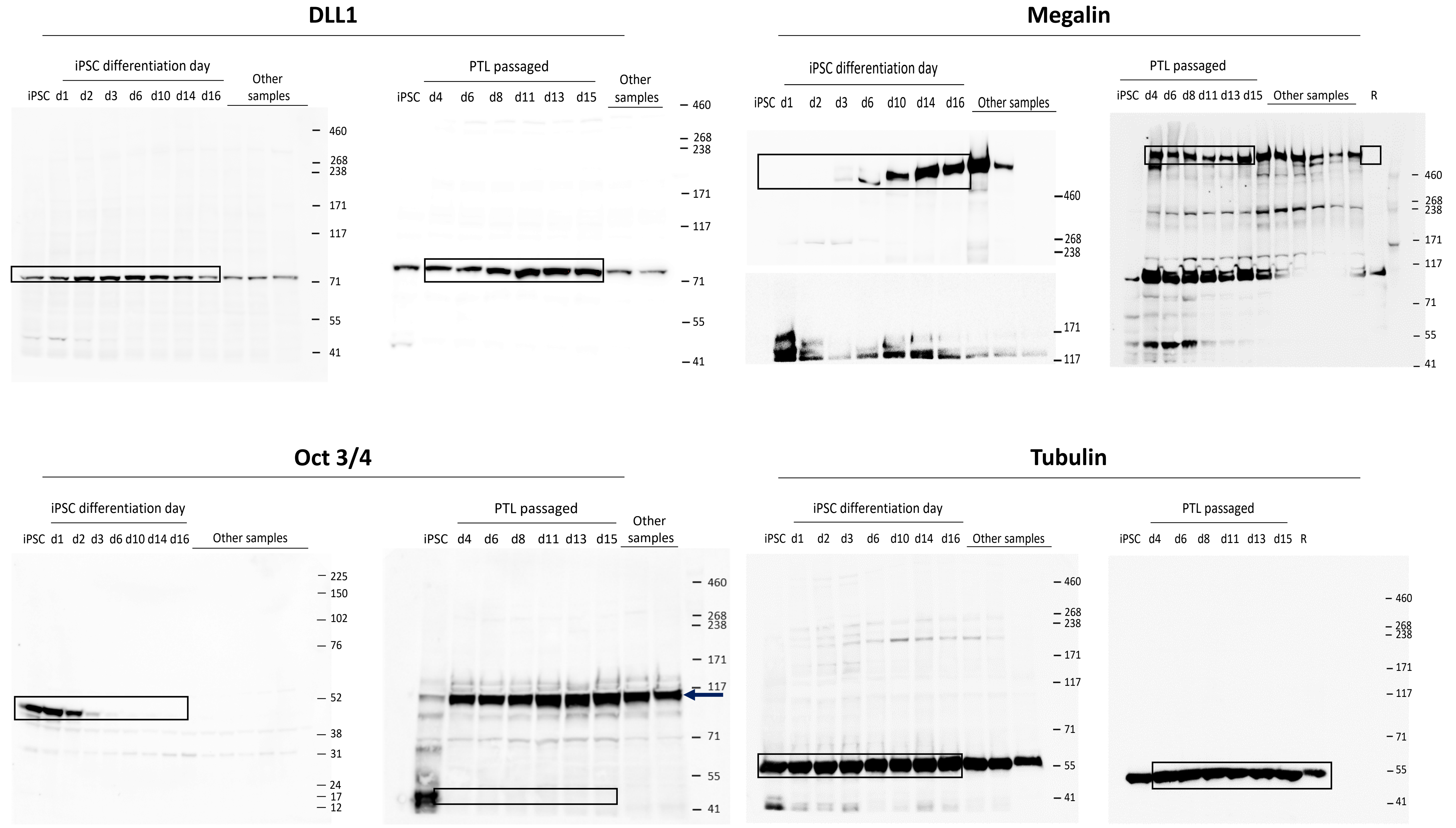

Figure S3C

SFC-086

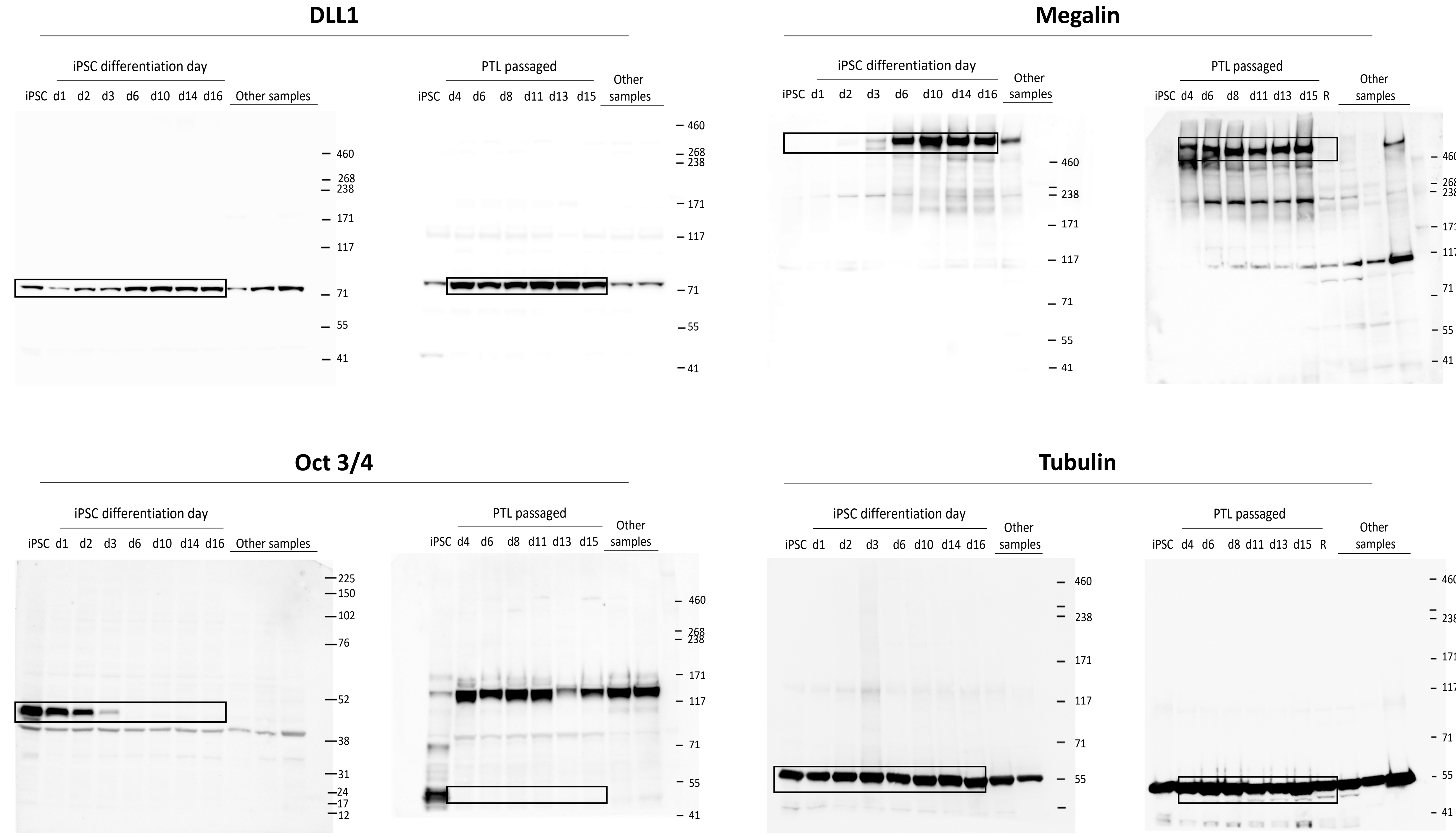

Figure S4

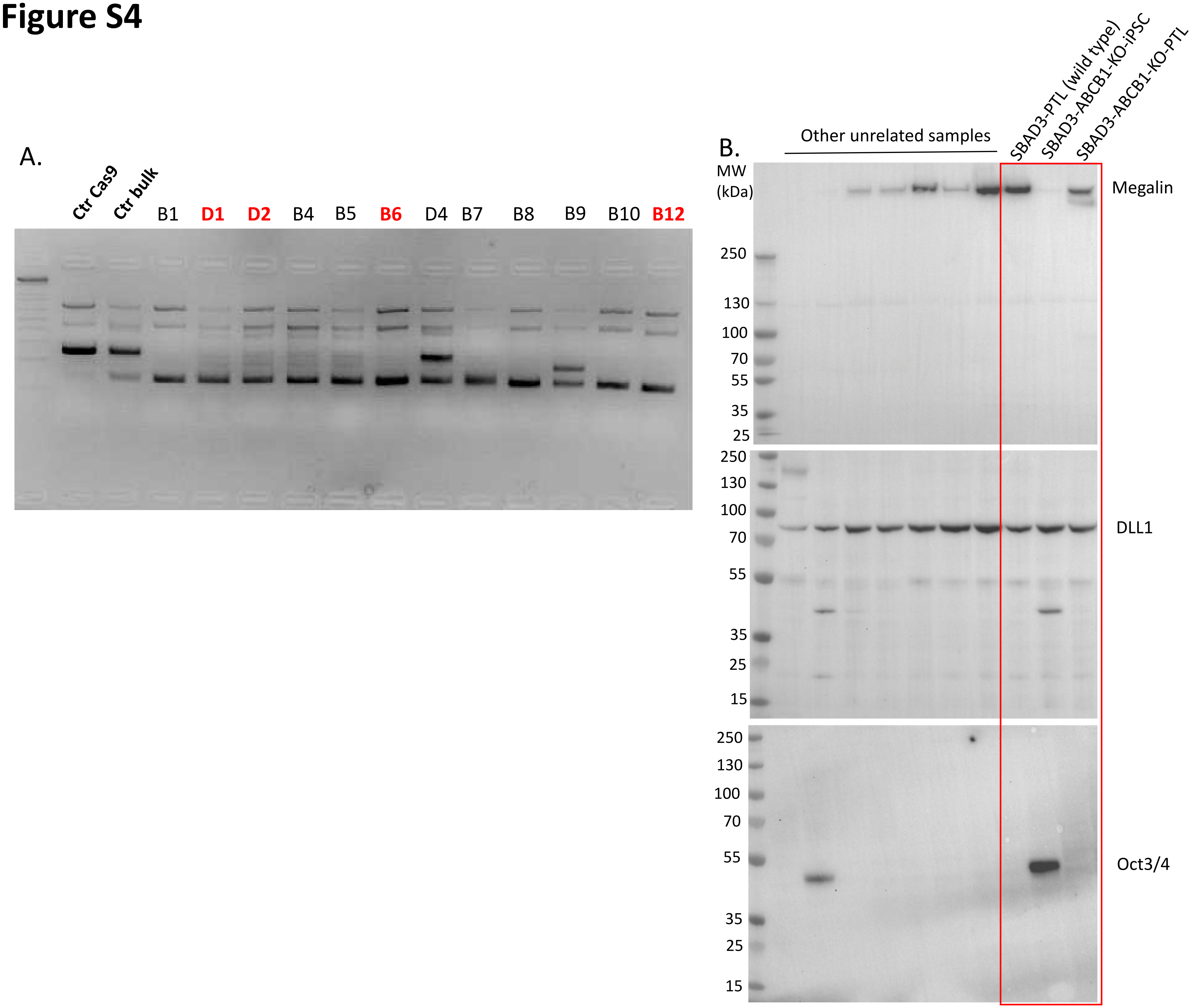

A.
Ctr Cas9
Ctr bulk
B1
D1
D2
B4
B5
B6
D4
B7
B8
B9
B10
B12
B.
Other unrelated samples
SBAD3-PTL (wild type)
SBAD3-ABCB1-KO-iPSC
SBAD3-ABCB1-KO-PTL
MW (kDa)
250
130
100
70
55
35
25
15
Megalin
DLL1
Oct3/4
